# Antiplatelet Therapy Discontinuation and the Risk of Serious Cardiovascular Events after Coronary Stenting: Observations from the CREDO-Kyoto Registry Cohort-2

**DOI:** 10.1371/journal.pone.0124314

**Published:** 2015-04-08

**Authors:** Hirotoshi Watanabe, Takeshi Morimoto, Masahiro Natsuaki, Yutaka Furukawa, Yoshihisa Nakagawa, Kazushige Kadota, Kyohei Yamaji, Kenji Ando, Satoshi Shizuta, Hiroki Shiomi, Tomohisa Tada, Junichi Tazaki, Yoshihiro Kato, Mamoru Hayano, Mitsuru Abe, Takashi Tamura, Manabu Shirotani, Shinji Miki, Mitsuo Matsuda, Mamoru Takahashi, Katsuhisa Ishii, Masaru Tanaka, Takeshi Aoyama, Osamu Doi, Ryuichi Hattori, Masayuki Kato, Satoru Suwa, Akinori Takizawa, Yoshiki Takatsu, Eiji Shinoda, Hiroshi Eizawa, Teruki Takeda, Jong-Dae Lee, Moriaki Inoko, Hisao Ogawa, Shuichi Hamasaki, Minoru Horie, Ryuji Nohara, Hirofumi Kambara, Hisayoshi Fujiwara, Kazuaki Mitsudo, Masakiyo Nobuyoshi, Toru Kita, Adnan Kastrati, Takeshi Kimura, CREDO-Kyoto PCI/CABG registry cohort-2 investigators

**Affiliations:** 1 Department of Cardiovascular Medicine, Graduate School of Medicine, Kyoto University, Kyoto, Japan; 2 Division of Genera Medicine, Department of Internal Medicine, Hyogo College of Medicine, Nishinomiya, Japan; 3 Department of Cardiovascular Medicine, Kobe City Medical Center General Hospital, Kobe, Japan; 4 Division of Cardiology, Tenri Hospital, Tenri, Japan; 5 Division of Cardiology, Kurashiki Central Hospital, Kurashiki, Japan; 6 Division of Cardiology, Kokura Memorial Hospital, Kitakyushu, Japan; 7 Division of Cardiology, National Hospital Organization Kyoto Medical Center, Kyoto, Japan; 8 Division of Cardiology, Japanese Red Cross Wakayama Medical Center, Wakayama, Japan; 9 Division of Cardiology, Nara Hospital, Kinki University Faculty of Medicine, Nara, Japan; 10 Division of Cardiology, Mitsubishi Kyoto Hospital, Kyoto, Japan; 11 Division of Cardiology, Kishiwada City Hospital, Kishiwada, Japan; 12 Division of Cardiology, Shimabara Hospital, Kyoto, Japan; 13 Division of Cardiology, Kansai Electric Power Hospital, Osaka, Japan; 14 Division of Cardiology, Osaka Red Cross Hospital, Osaka, Japan; 15 Division of Cardiology, Shimada Municipal Hospital, Shimada, Japan; 16 Division of Cardiology, Shizuoka General Hospital, Shizuoka, Japan; 17 Division of Cardiology, Maizuru Kyosai Hospital, Maizuru, Japan; 18 Division of Cardiology, Juntendo University Shizuoka Hospital, Shizuoka, Japan; 19 Division of Cardiology, Shizuoka City Shizuoka Hospital, Shizuoka, Japan; 20 Division of Cardiology, Hyogo Prefectural Amagasaki Hospital, Amagasaki, Japan; 21 Division of Cardiology, Hamamatsu Rosai Hospital, Hamamatsu, Japan; 22 Division of Cardiology, Nishi-Kobe Medical Center, Kobe, Japan; 23 Division of Cardiology, Koto Memorial Hospital, Higashioumi, Japan; 24 Division of Cardiology, University of Fukui Hospital, Fukui, Japan; 25 Cardiovascular Center, the Tazuke Kofukai Medical Research Institute, Kitano Hospital, Osaka, Japan; 26 Department of Cardiovascular Medicine, Graduate School of Medical Sciences, Kumamoto University, Kumamoto, Japan; 27 Department of Cardiovascular, Respiratory and Metabolic Medicine, Graduate School of Medicine, Kagoshima University, Kagoshima, Japan; 28 Department of Cardiovascular and Respiratory, Shiga University of Medical Science, Otsu, Japan; 29 Division of Cardiology, Hirakata Kohsai Hospital, Hirakata, Japan; 30 Deutsches Herzzentrum, Munich, Germany; University Hospital Medical Centre, GERMANY

## Abstract

Relation of antiplatelet therapy (APT) discontinuation with the risk of serious cardiovascular events has not been fully addressed yet. This study is aimed to evaluate the risk of ischemic event after APT discontinuation based on long-term APT status of large cohort. In the CREDO-Kyoto Registry Cohort-2 enrolling 15939 consecutive patients undergoing first coronary revascularization, 10470 patients underwent percutaneous coronary intervention either with bare-metal stents (BMS) only (N=5392) or sirolimus-eluting stents (SES) only (N=5078). Proportions of patients taking dual-APT were 67.3% versus 33.4% at 1-year, and 48.7% versus 24.3% at 5-year in the SES and BMS strata, respectively. We evaluated daily APT status (dual-, single- and no-APT) and linked the adverse events to the APT status just 1-day before the events. No-APT as compared with dual- or single-APT was associated with significantly higher risk for stent thrombosis (ST) beyond 1-month after SES implantation (cumulative incidence rates beyond 1-month: 1.23 versus 0.15/0.29, P<0.001/P<0.001), while higher risk of no-APT for ST was evident only until 6-month after BMS implantation (incidence rates between 1- and 6-month: 8.43 versus 0.71/1.20, P<0.001/P<0.001, and cumulative incidence rates beyond 6-month: 0.31 versus 0.11/0.08, P=0.16/P=0.08). No-APT as compared with dual- or single-APT was also associated with significantly higher risk for spontaneous myocardial infarction (MI) and stroke regardless of the types of stents implanted. Single-APT as compared with dual-APT was not associated with higher risk for serious adverse events, except for the marginally higher risk for ST in the SES stratum. In conclusion, discontinuation of both aspirin and thienopyridines was associated with increased risk for serious cardiovascular events including ST, spontaneous MI and stroke beyond 1-month after coronary stenting.

## Introduction

Early reports suggested that premature discontinuation of dual antiplatelet therapy (APT) was the most potent predictor of stent thrombosis (ST) after drug-eluting stent (DES) implantation [1–6]. However, evaluating the influence of APT discontinuation on cardiovascular events is quite complex because of the diversity including timing of discontinuation after coronary stent implantation, reasons for discontinuation, mode of APT after discontinuation, duration of discontinuation, and restart of APT. Many previous reports evaluating the impact of APT discontinuation on ST could not fully address these complex features of APT discontinuation [1–20]. Furthermore, there was only 1 previous report that systemically evaluated the impact of APT discontinuation after coronary stenting on spontaneous myocardial infarction (MI) [18] and no report for stroke. Therefore, in an attempt to understand further the influences of APT discontinuation on serious cardiovascular events after coronary stenting, we linked daily status of APT administration (both aspirin and thienopyridines) to subsequent ST, spontaneous MI, and stroke utilizing the long-term follow-up data from a large Japanese observational registry of patients who underwent sirolimus-eluting stents (SES) or bare-metal stents (BMS) implantation.

## Materials and Methods

### Study Design and Population

**Fig 1:**
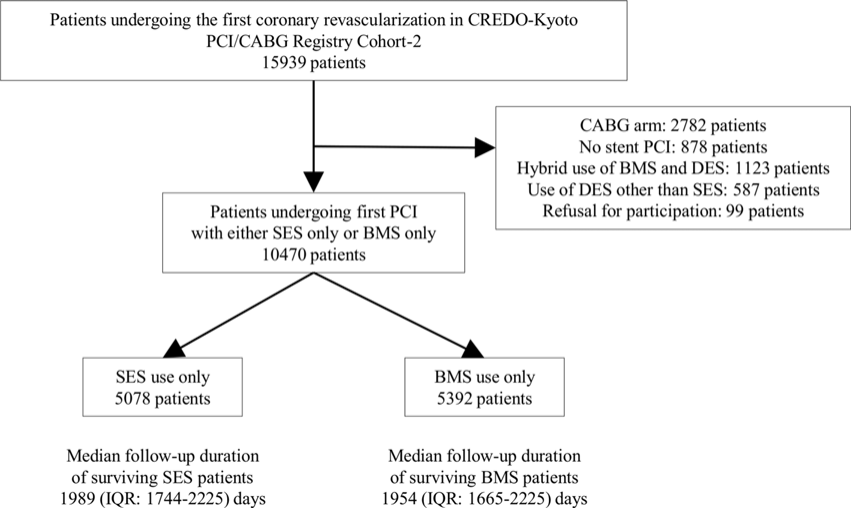
Study Flow. BMS = bare-metal stents, CABG = coronary artery bypass grafting, CREDO-Kyoto = Coronary REvascularization Demonstrating Outcome study in Kyoto, DES = drug-eluting stent, IQR = interquartile range, PCI = percutaneous coronary intervention, and SES = sirolimus-eluting stents.

The Coronary REvascularization Demonstrating Outcome study in Kyoto (CREDO-Kyoto) percutaneous coronary intervention (PCI)/coronary artery bypass grafting (CABG) registry cohort-2 is a physician-initiated non-company sponsored multicenter registry enrolling consecutive patients undergoing first coronary revascularization procedures at 26 centers in Japan from January 2005 to December 2007 (S1 Appendix). The design and patient enrollment of the registry has been described previously [21]. Among 15939 study patients, 10470 patients were included in the current analysis, including 5078 patients treated with SES only and 5392 patients treated with BMS only (Fig 1). We recently reported the long-term clinical outcome of SES as compared with BMS in this registry [22]. In the current analysis, we evaluated the relation between APT discontinuation and serious cardiovascular events in the SES and BMS strata. The recommended APT regimen was aspirin (> = 81mg daily) indefinitely and thienopyridine (200 mg ticlopidine or 75 mg clopidogrel daily) for at least 3 months after SES implantation, and for at least 1 month after BMS implantation.

### Ethics

The research protocol (S1 Protocol) was approved by the local ethics committees in all of 26 participating medical centers (Kyoto University Hospital, Kishiwada City Hospital, Tenri Hospital, Hyogo Prefectural Amagasaki Hospital, Kitano Hospital, Koto Memorial Hospital, Kokura Memorial Hospital, Maizuru Kyosai Hospital, Nara Hospital Kinki University Faculty of Medicine, Kobe City Medical Center General Hospital, Nishi-Kobe Medical Center, Kansai Denryoku Hospital, Osaka Red Cross Hospital, University of Fukui Hospital, Shizuoka City Shizuoka Hospital, Hamamatsu Rosai Hospital, Shiga University of Medical Science Hospital, Japanese Red Cross Wakayama Medical Center, Shimabara Hospital, Kagoshima University Medical and Dental Hospital, Shizuoka General Hospital, Kurashiki Central Hospital, Mitsubishi Kyoto Hospital, Kumamoto University Hospital, Shimada Municipal Hospital, and Juntendo University Shizuoka Hospital). Because of retrospective enrollment, written informed consents from the patients are waived; however, we exclude those patients who refuse participation in the study when contacted for follow-up. Patient records was anonymized and de-identified prior to analysis.

### Study Endpoints

The outcome measures for the current analysis were Academic Research Consortium definite ST, spontaneous MI, and stroke [23]. Spontaneous MI was defined as type 1 in the universal classification [24]. Stroke was defined as ischemic or hemorrhagic stroke either occurring during the index hospitalization or requiring hospitalization with symptoms lasting >24 hours.

### Data Collection

Follow-up data on clinical events together with APT status were collected by the experienced clinical research coordinators in the independent clinical research organization (Research Institute for Production Development, Kyoto, Japan; S1 Appendix). ST, spontaneous MI, and stroke were adjudicated by a clinical event committee (S1 Appendix). Data on APT status during follow-up was collected from the hospital charts in the participating centers (72.3%), letters to patients (19.9%), and telephone call to referring physicians (6.5%). Dates of discontinuation of aspirin and thienopyridine including temporary and permanent discontinuation were reported separately. If the drug was restarted after discontinuation, the dates of restart were also recorded. If we could not identify the exact date of discontinuation in patients not on the drug at follow-up, we adopted the median date between the last day known to have continued the drug and the day of follow-up (8.3% of all discontinuations). Similarly, if we could not identify the exact date of restart in patients on the drug at follow-up, we adopted the median date between the day of discontinuation and the day of follow-up (0.4% of all restart).

### Statistical Analysis

**Fig 2:**
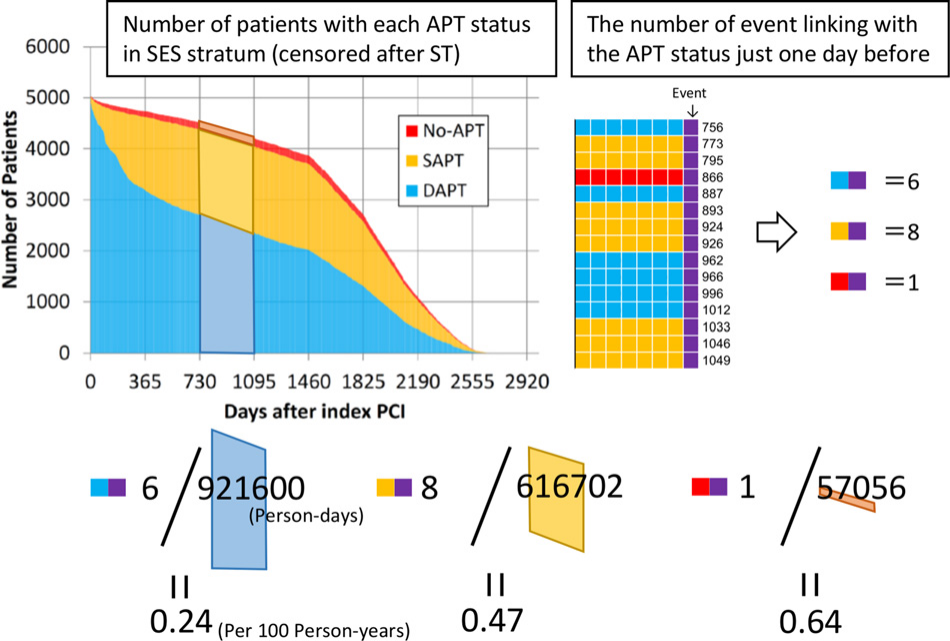
Schematic Illustration for the Actual Method Calculating the Incidence Rate of Events for Each APT status. As an example, this figure illustrates how to calculate the incidence rate of stent thrombosis under dual-APT during the interval of 731–1095 days after index SES implantation. For the calculation of incident rate, the numerator is the number of events in patients on dual-APT just 1-day before the event, and the denominator is the total person-days on dual-APT, which is censored after event and expressed as trapezoidal area in the graph. Incidence rate was presented in 100 person-years units. APT = antiplatelet therapy, DAPT = dual-APT, PCI = percutaneous coronary intervention, SES = sirolimus-eluting stents, and ST = stent thrombosis.

We coded the APT status on daily basis and classified the daily APT status as dual-APT, aspirin only, thienopyridine only, and no-APT. If a serious cardiovascular event occurred, this event was linked to the APT status just 1-day before the event. We did not choose the APT status on the day of the event, because it could be influenced by the event. Patients who experienced the endpoint events were censored on the day of the events. Observations without information of APT status 1-day before the corresponding event were excluded from the analysis. Dividing the number of events by the number of patients in each APT status throughout the pre-specified time intervals after the index stent implantation (within 30 days, 31–180 days, 181–365 days, 366–730 days, 731–1095 days, 1096–1460 days, 1461–1825 days, 1826–2190 days, and 2195–2555 days), we estimated the incidence rate during those time intervals. We expressed the incidence rate as the number of events per 100 person-years (Fig 2). Cumulative incidence rate was calculated in the interval from the pre-specified landmark points (31-day, 181-day, 366-day, 731-day, 1096-day, 1461-day, 1826-day, and 2195-day) to the end of follow-up (2788-day).

Categorical variables were expressed as number and percentages. Continuous variables were expressed as mean +/- SD or median and interquartile range. In the comparison of incidence rates among each APT status, we used the likelihood-ratio chi-square test.

To identify prognostically important factors other than daily APT status, we constructed cox regression models for each endpoint including 39 variables used in our previous report [22]. Based on the results of the cox models, we chose the following 8 variables as risk adjusting variables: age > = 75, male gender, diabetes mellitus, multivessel disease, previous stroke, previous atrial fibrillation, renal dysfunction, and target of proximal left anterior descending coronary artery (S1 Table). We then developed the logistic regression models to estimate the risk (odds ratio) of no-APT and single-APT relative to dual-APT about adverse event beyond 1-month after index PCI adjusting for the 8 variables described above, assuming that all observations (patient-day) were independent because our interest was to assess the association between daily APT status and the events on the next day.

The duration from APT discontinuation to the onset of adverse event was compared between the single-APT and no-APT groups, using Wilcoxon signed-rank test.

Analysis was made by using JMP 10.0. (SAS Institute Inc., Cary, NC, USA), and Excel 2010 (Microsoft Co., Redmond, WA, USA). P value <0.05 was considered as statistically significant.

## Results

### Baseline Characteristics and Clinical Outcomes through 7-Year

Baseline characteristics were previously described [21]. In brief, the SES stratum as compared with the BMS stratum included fewer patients with acute myocardial infarction presentation and more patients with complex clinical and angiographic characteristics (Table 1). Most of the patients received ticlopidine rather than clopidogrel as the thienopyridine, because in Japan the indication of clopidogrel for coronary stent implantation was approved in October 2007, near the end of the study patient enrollment. Clinical outcome through 7-year indicated that the cumulative incidences of ST and spontaneous MI were similar between the SES and BMS strata, and stroke was the more frequently encountered cardiovascular event than ST and spontaneous MI (Table 2).

**Table 1:** Baseline Clinical and Procedural Characteristics and Medications.

	SES stratum	BMS stratum
	(N = 5078)	(N = 5392)
**Clinical Characteristics**		
Age, years	68.4±10.4	67.9±11.8
Age > = 75 years	1547 (31%)	1717 (32%)
Male	3624 (71%)	3922 (73%)
BMI	23.8±3.4	23.7±3.5
BMI <25.0	3408 (67%)	3745 (69%)
Hypertension	4212 (83%)	4364 (81%)
Diabetes mellitus	2069 (41%)	1787 (33%)
Diabetes with insulin therapy	520 (10%)	255 (4.7%)
Current smoking	1372 (27%)	1988 (37%)
Acute myocardial infarction	820 (16%)	2890 (54%)
Heart failure	875 (17%)	1182 (22%)
Shock at presentation	114 (2.2%)	499 (9.3%)
Multivessel disease	2966 (58%)	2420 (45%)
Mitral regurgitation grade 3/4	164 (3.2%)	230 (4.3%)
LVEF, %	59.9±12.9	57.8±13.1
Previous myocardial infarction	685 (14%)	382 (7.1%)
Previous stroke	579 (11%)	506 (9.4%)
Peripheral vascular disease	403 (7.9%)	394 (7.3%)
eGFR <30 ml/min/1.73m^2^, not on dialysis	198 (3.9%)	234 (4.3%)
Dialysis	228 (4.5%)	116 (2.2%)
Atrial Fibrillation	404 (8.0%)	489 (9.1%)
Anemia (Hemoglobin <11 g/dl)	582 (12%)	614 (11%)
Thrombocytopenia (Platelet <100 10^9^/L)	72 (1.4%)	79 (1.5%)
COPD	172 (3.4%)	211 (3.9%)
Liver cirrhosis	116 (2.3%)	154 (2.9%)
Malignancy	441 (8.7%)	539 (10%)
**Procedural Characteristics**		
Number of target lesions	1.47±0.74	1.30±0.62
Target of proximal LAD	3159 (62%)	2804 (52%)
Target of unprotected LMCA	172 (3.4%)	154 (2.9%)
Target of CTO	673 (13%)	339 (6.3%)
Target of bifurcation,	1981 (39%)	1217 (23%)
Bifurcation with side-branch stenting,	246 (4.8%)	127 (2.4%)
Total number of stents	1.90±1.22	1.46±0.82
Total stent length, mm	42.3±30.2	29.1±18.8
Total stent length >28mm	2674 (53%)	1804 (34%)
Minimum stent size, mm	2.83±0.37	3.09±0.49
Minimum stent size <3.0mm	2538 (50%)	1576 (29%)
**Baseline medication**		
Antiplatelet therapy		
Thienopyridine	5062 (99.7%)	5288 (98%)
Ticlopidine	4503 (89%)	4887 (91%)
Clopidogrel	551 (11%)	380 (7.0%)
Aspirin	5010 (99%)	5318 (99%)
Cilostazole	791 (16%)	1204 (22%)
Other medications		
Statins	2737 (54%)	2594 (48%)
Beta-blockers	1418 (28%)	1717 (32%)
ACE-I/ARB	2797 (55%)	3259 (60%)
Nitrates	1882 (37%)	1789 (33%)
Calcium channel blockers	2369 (47%)	1858 (35%)
Nicorandil	1087 (21%)	1350 (25%)
Warfarin	413 (8.1%)	404 (7.5%)
Proton pump inhibitors	1183 (23%)	1498 (28%)
H2-blockers	1198 (24%)	1515 (28%)

ACE-I = angiotensin converting enzyme inhibitors, ARB = angiotensin II receptor blockers, BMI = body mass index, BMS = bare-metal stents, COPD = chronic obstructive pulmonary disease, CTO = chronic total occlusion, eGFR = estimated glomerular filtration rate, LAD = left anterior descending coronary artery, LMCA = left main coronary artery, LVEF = left ventricular ejection fraction, and SES = sirolimus-eluting stents.

**Table 2:** Clinical Outcomes through 7-Year.

	Number of patients with event (Cumulative Incidence)	
	SES stratum	BMS stratum
	N = 5078	N = 5392
Death	847 (23.7%)	1064 (25.8%)
Cardiac	364 (10.5%)	515 (11.9%)
Non-cardiac	483 (14.7%)	549 (15.9%)
All MI	341 (9.0%)	372 (9.1%)
MI excluding non-Q MI within 7 days	213 (6.5%)	268 (7.0%)
Spontaneous MI	83 (3.1%)	128 (3.8%)
Definite ST	73 (1.8%)	108 (2.5%)
All Stroke	334 (9.2%)	309 (7.8%)
Hemorrhagic stroke	91 (2.7%)	61 (1.9%)
Ischemic stroke	253 (6.8%)	251 (6.0%)
GUSTO severe bleeding	243 (6.5%)	251 (6.6%)

The cumulative incidences were calculated by Kaplan-Meier method.

GUSTO = global utilization of streptokinase and tissue plasminogen activator for occluded coronary arteries, MI = myocardial infarction, and ST = stent thrombosis.

### APT Status During Follow-Up

**Fig 3:**
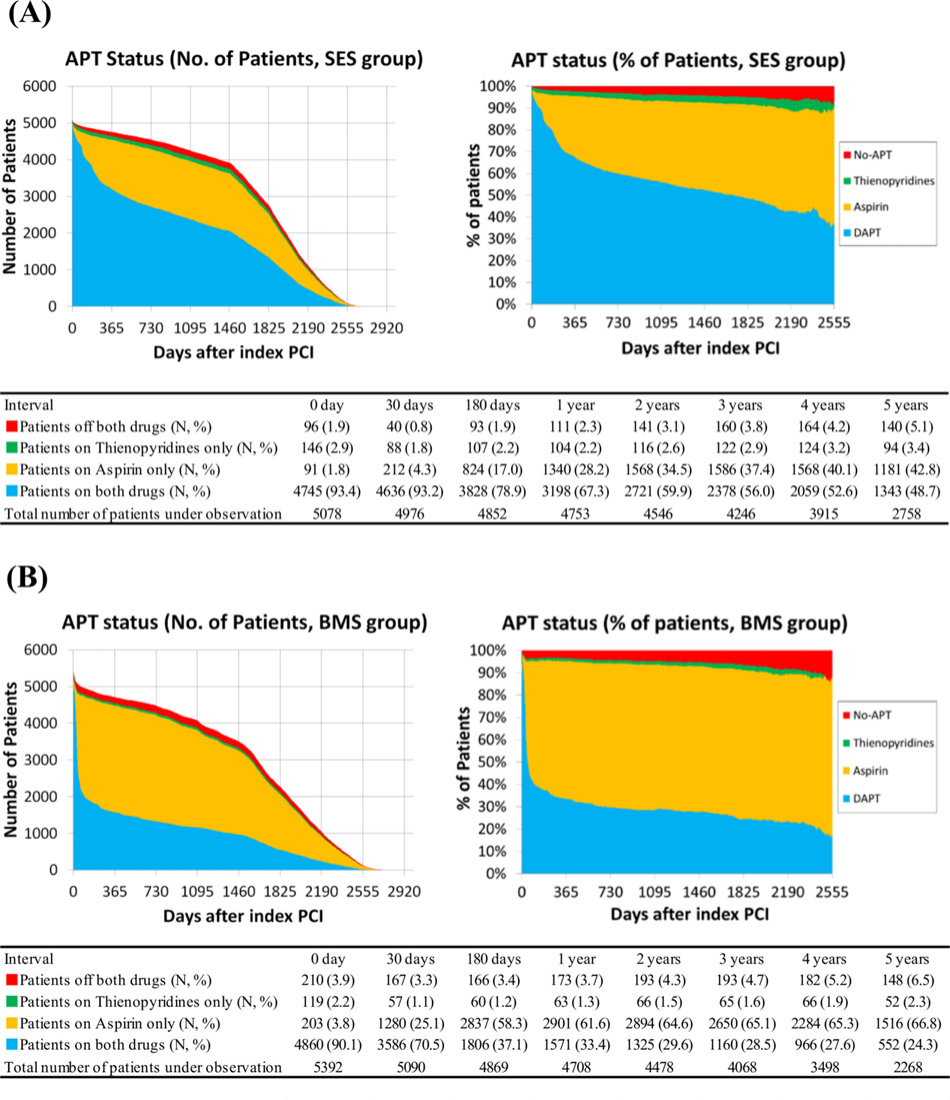
Prevalence of Each APT Status during Follow-Up. Prevalence of each APT status during follow-up in the SES group (A), and in the BMS group (B). APT = antiplatelet therapy, BMS = bare-metal stents, DAPT = dual-APT, PCI = percutaneous coronary intervention, and SES = sirolimus-eluting stents.

The proportion of patients receiving dual-APT was higher in the SES stratum than in the BMS stratum (93.2% versus 70.5% at 30-day, 67.3% versus 33.4% at 1-year, and 48.7% versus 24.3% at 5-year, respectively) (Fig 3). The rate of dual-APT continuation in the SES stratum remained high (52.0% at 5-year) even after exclusion of those patients with other indications of prolonged dual-APT such as prior stroke and peripheral vascular disease. When patients with repeated PCI during follow-up were censored at the time of repeated PCI, the differences in the proportion of patients receiving dual-APT between the SES and BMS strata became greater (64.5% versus 24.5% at 1-year, and 40.2% versus 14.3% at 5-year, respectively) (S1 Fig).

The proportion of no-APT patients was small, but increased during follow-up in both SES and BMS strata (0.8% versus 3.3% at 30-day, 2.3% versus 3.7% at 1-year, and 5.1% versus 6.5% at 5-year, respectively) (Fig 3).

### Definite ST

**Fig 4:**
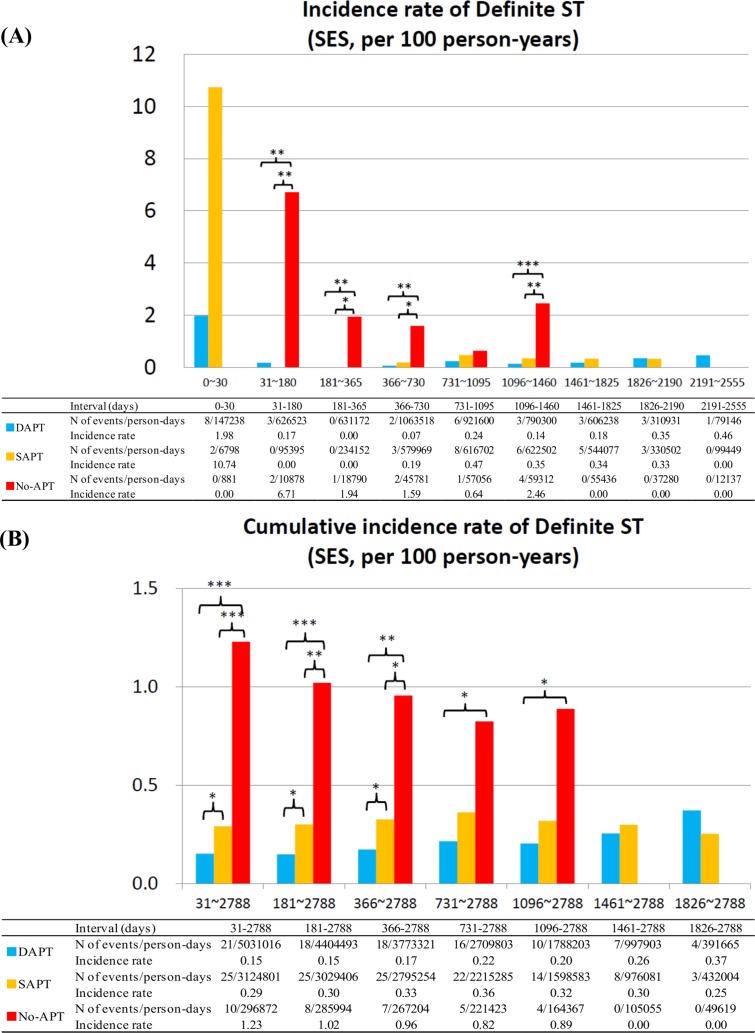
Incidence Rates for Definite ST in the SES group. (A) Incidence rates of definite ST in the SES group in the pre-specified time intervals, and (B) cumulative incidence rates of definite ST in the SES group. *: P <0.05, **: P <0.01; and ***: P <0.001. APT = antiplatelet therapy, DAPT = dual-APT, SAPT = single-APT, SES = sirolimus-eluting stents, and ST = stent thrombosis.

In the SES stratum, the incidence rates for ST tended to be higher in no-APT patients as compared with dual- or single-APT patients in all the time intervals between 31 days and 1460 days. Cumulative incidence rate for ST beyond 1-month was significantly higher in no-APT patients as compared to patients with dual- or single-APT (Fig 4). Therefore, discontinuation of both aspirin and thienopyridines was associated with significantly higher risk for ST beyond 1-month and up to 4-year after SES implantation.

**Fig 5:**
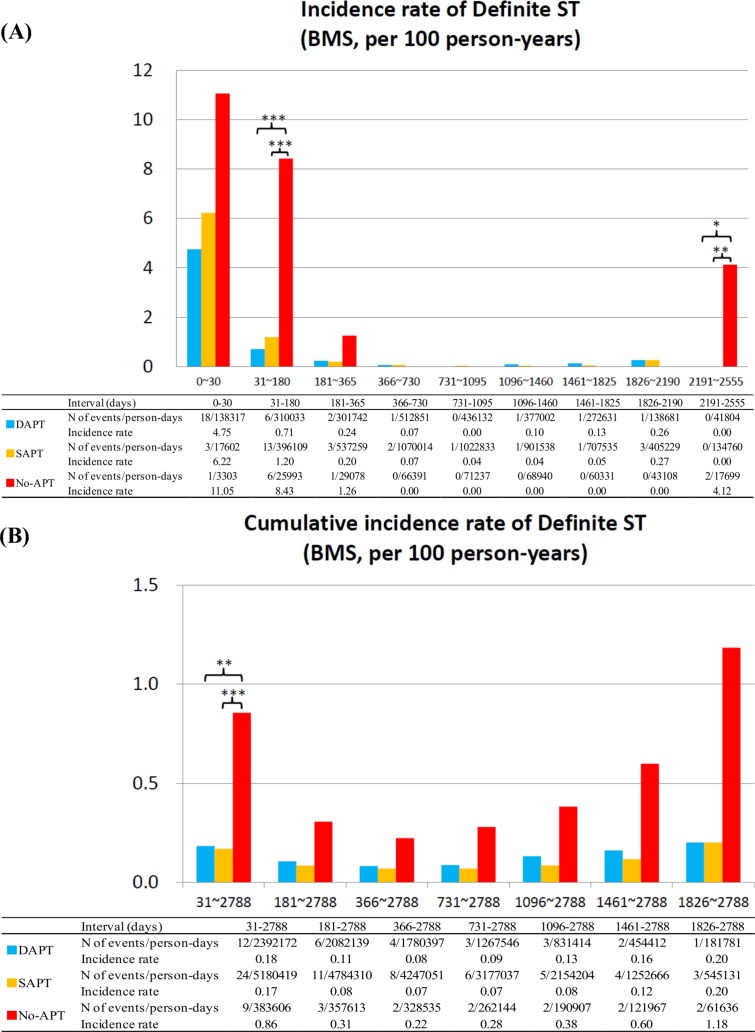
Incidence Rates for Definite ST in the BMS group. (A) Incidence rates of definite ST in the BMS group in the pre-specified time intervals, and (B) cumulative incidence rates of definite ST in the BMS group. *: P <0.05, **: P <0.01; and ***: P <0.001. APT = antiplatelet therapy, BMS = bare-metal stents, DAPT = dual-APT, SAPT = single-APT, and ST = stent thrombosis.

In the BMS stratum, higher risk for ST in no-APT patients was evident only in the interval of 31–180 days. Incidence rates of ST were very low regardless of APT status in all the time intervals between 181 days and 2190 days. Cumulative incidence rate of ST beyond 6-month in no-APT patients was not significantly different from that in dual- or single APT patients (Fig 5).

In both the SES and BMS strata, incidence rates for ST were not significantly different between dual-APT and single-APT in any time intervals beyond 1-month. However, single-APT as compared with dual-APT after SES implantation, but not after BMS implantation, was associated with higher cumulative incidence rate for ST beyond 1-month (Figs 4 and 5). The duration from APT discontinuation to the onset of ST was markedly shorter in the no-APT group than in the single-APT group (Table 3; S2 Fig).

**Table 3:** Duration from Drug Discontinuation to the Onset of Adverse Events.

	SAPT	No-APT	
	Median (IQR) days	Median (IQR) days	P value
SES stratum			
Definite ST	949 (451–1731)	36.5 (8–632)	<0.001
Spontaneous MI	749 (203–1392)	277 (20–750)	0.09
Stroke	519 (137–976)	30 (7–124)	<0.001
BMS stratum			
Definite ST	100 (37–640)	18.5 (8–36)	0.005
Spontaneous MI	721 (277–1327)	473 (14–778)	0.04
Stroke	625 (222–1141)	44 (5–840)	0.001

Duration of SAPT is the number of days from the last day when patients received DAPT to the day of adverse event, without any consideration for days with no-APT in between, if any, for the patients who had adverse event on SAPT. Duration of no-APT is the number of consecutive days with no-APT just before the adverse event.

APT = antiplatelet therapy, BMS = bare-metal stents, DAPT = dual-APT, IQR = interquartile range, MI = myocardial infarction, SAPT = single-APT, SES = sirolimus-eluting stents, and ST = stent thrombosis.

### Spontaneous MI

**Fig 6:**
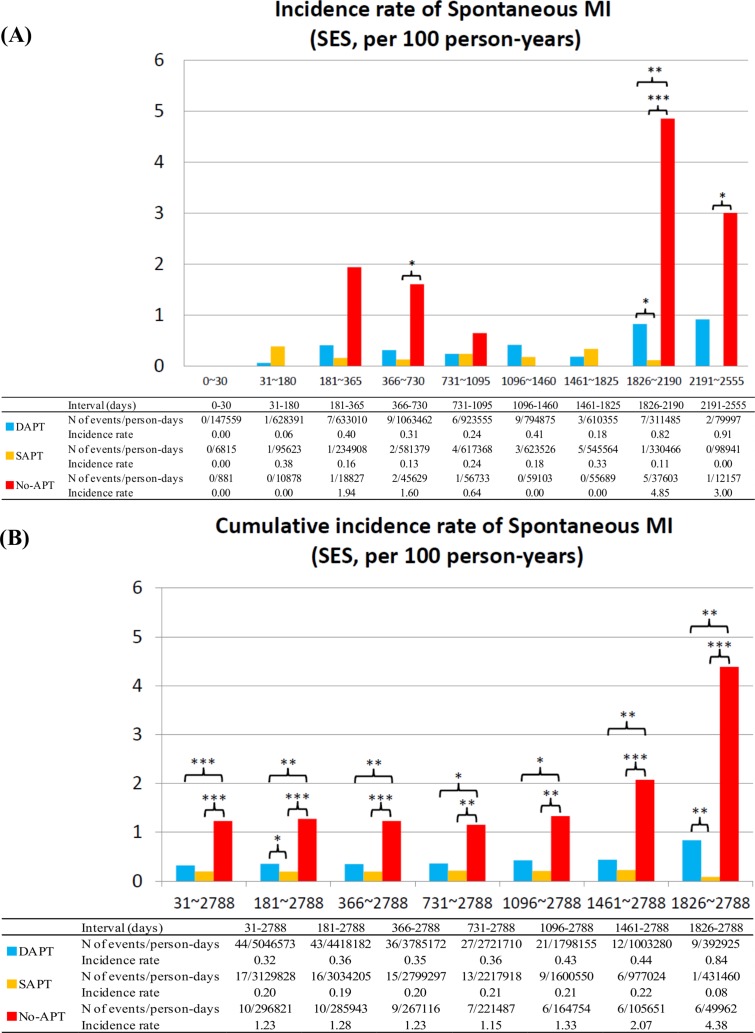
Incidence Rates for Spontaneous MI in the SES group. (A) Incidence rates of spontaneous MI in the SES group in the pre-specified time intervals, and (B) cumulative incidence rates of spontaneous MI in the SES group. APT = antiplatelet therapy, DAPT = dual-APT, MI = myocardial infarction, SAPT = single-APT, SES = sirolimus-eluting stents, and ST = stent thrombosis.

**Fig 7:**
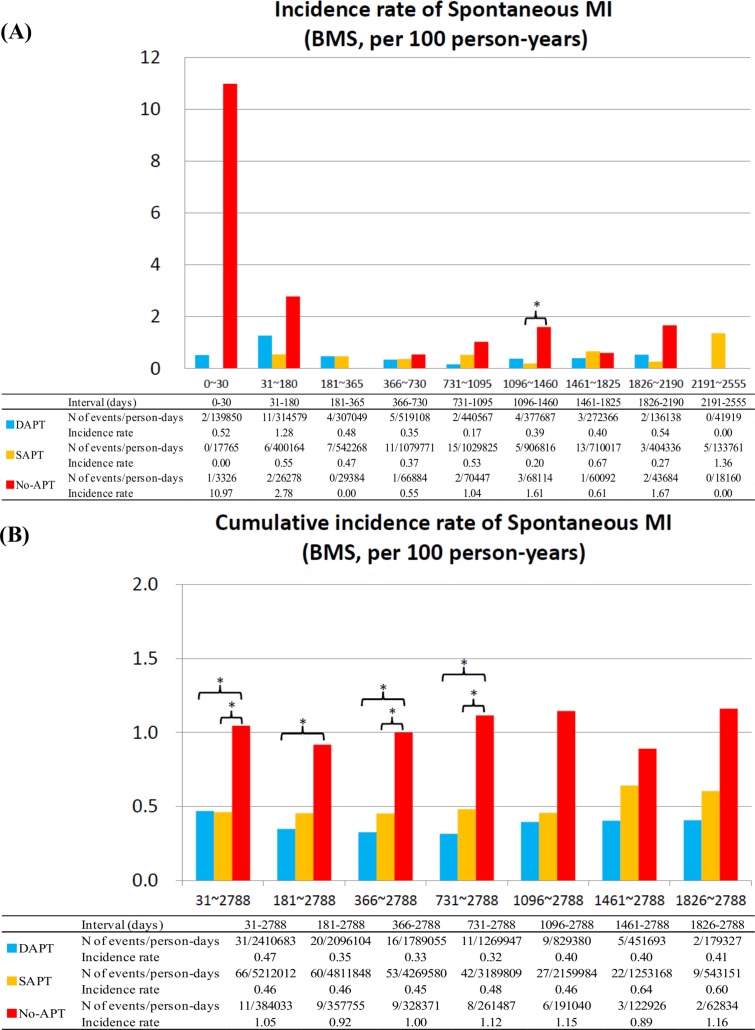
Incidence Rates for Spontaneous MI in the BMS group. (A) Incidence rates of spontaneous MI in the BMS group in the pre-specified time intervals, and (B) cumulative incidence rates of spontaneous MI in the BMS group. APT = antiplatelet therapy, BMS = bare-metal stents, DAPT = dual-APT, MI = myocardial infarction, SAPT = single-APT, and ST = stent thrombosis.

No-APT as compared with dual- or single-APT was associated with higher risk for spontaneous MI, while single-APT as compared with dual-APT was not associated with higher risk for spontaneous MI regardless of the timing after stent implantation and regardless of the stent types implanted (Figs 6 and 7). The duration from APT discontinuation to the onset of spontaneous MI in SES strata tended to be shorter in the no-APT group than in the single-APT group, although in BMS strata the duration was significantly shorter in no-APT group (Table 3; S3 Fig)

### Stroke

**Fig 8:**
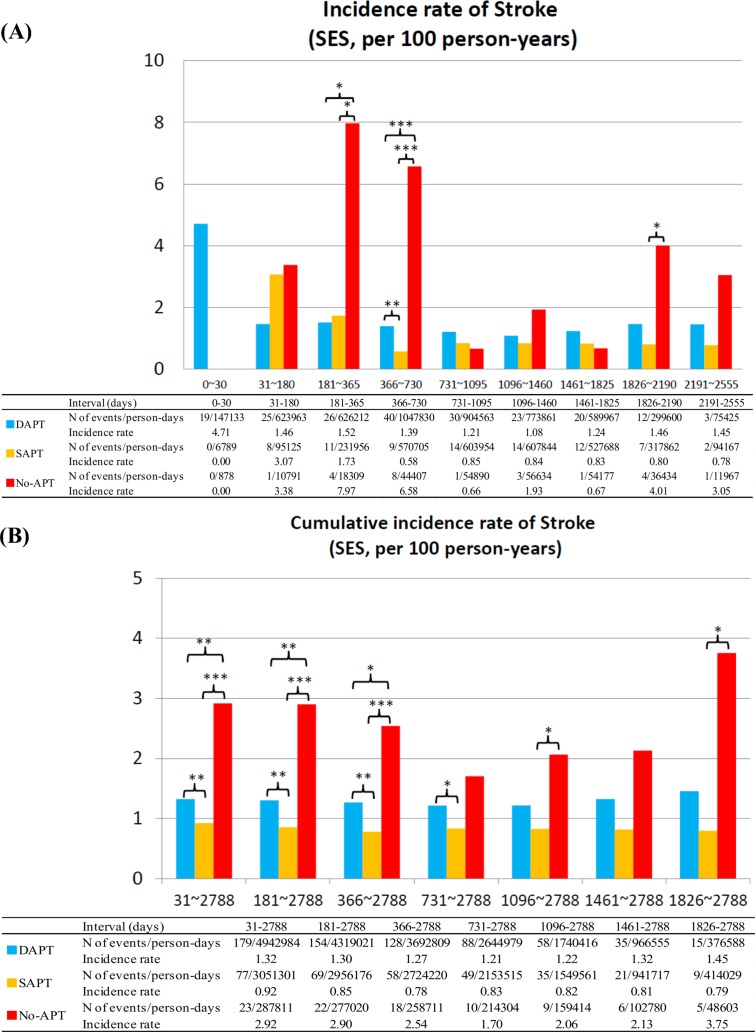
Incidence Rates for Stroke in the SES group. (A) Incidence rates of stroke in the SES group in the pre-specified time intervals, and (B) cumulative incidence rates of stroke in the SES group. APT = antiplatelet therapy, DAPT = dual-APT, SAPT = single-APT, SES = sirolimus-eluting stents, and ST = stent thrombosis.

**Fig 9:**
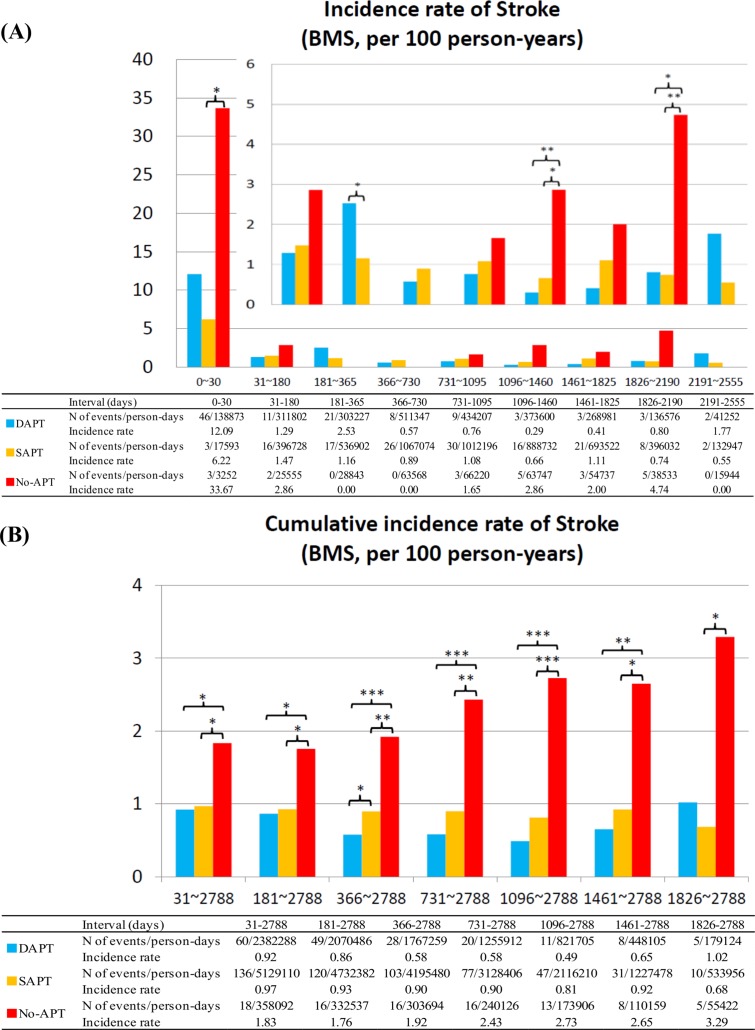
Incidence Rates for Stroke in the BMS group. (A) Incidence rates of stroke in the BMS group in the pre-specified time intervals, and (B) cumulative incidence rates of stroke in the BMS group. APT = antiplatelet therapy, BMS = bare-metal stents, DAPT = dual-APT, SAPT = single-APT, and ST = stent thrombosis.

No-APT as compared with dual- or single-APT was also associated with higher risk for stroke, while single-APT as compared with dual-APT was not associated with higher risk for stroke regardless of the timing after stent implantation and regardless of the stent types implanted (Figs 8 and 9). The duration from APT discontinuation to the onset of stroke was also markedly shorter in the no-APT group than in the single-APT group (Table 3; S4 Fig). For all the 3 outcome measures, the adjusted results by multivariable logistic regression analysis were fully consistent with the unadjusted results (Table 4).

**Table 4:** Unadjusted and Adjusted Risk of No-APT and SAPT Relative to DAPT for Serious Adverse Events beyond 1-month after index PCI.

		Unadjusted OR	(95%CI)	P value	Adjusted OR	(95%CI)	P Value
**(A) Definite ST**							
SES stratum	SAPT	1.92	(1.07–3.45)	0.03	2.01	(1.12–3.61)	0.02
	No-APT	8.07	(3.64–16.72)	<0.001	9.54	(4.46–20.41)	<0.001
BMS stratum	SAPT	0.92	(0.47–1.91)	0.82	0.89	(0.45–1.80)	0.75
	No-APT	4.68	(1.91–11.05)	0.001	4.57	(1.91–10.93)	0.001
**(B) Spontaneous MI**							
SES stratum	SAPT	0.62	(0.35–1.07)	0.09	0.64	(0.37–1.13)	0.12
	No-APT	3.86	(1.84–7.36)	<0.001	3.65	(1.82–7.30)	<0.001
BMS stratum	SAPT	0.98	(0.65–1.53)	0.94	1.10	(0.72–1.69)	0.67
	No-APT	2.23	(1.07–4.30)	0.03	2.29	(1.15–4.58)	0.02
**(C) Stroke**							
SES stratum	SAPT	0.70	(0.53–0.91)	0.007	0.75	(0.57–0.99)	0.04
	No-APT	2.21	(1.39–3.33)	0.001	2.10	(1.36–3.25)	0.001
BMS stratum	SAPT	1.05	(0.78–1.44)	0.74	1.19	(0.88–1.62)	0.27
	No-APT	2.00	(1.15–3.31)	0.02	1.91	(1.12–3.24)	0.02

APT = no antiplatelet therapy, BMS = bare-metal stents, CI = confidence interval, DAPT = dual-APT, OR = odds ratio, SAPT = single-APT, and SES = sirolimus-eluting stents.

### APT Status During 30 Days Before Onset of Definite ST

**Fig 10:**
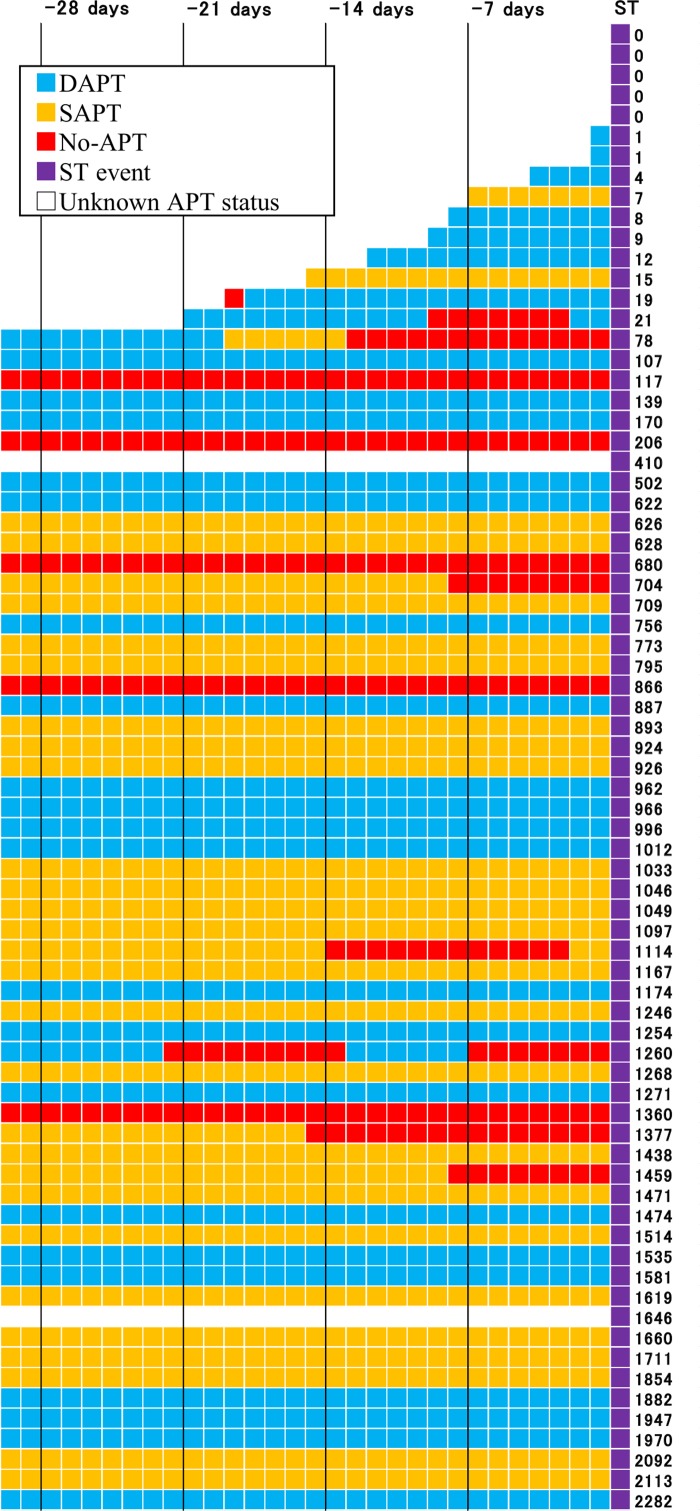
APT Status during 30 days Before Stent Thrombosis in the SES Group. Numbers on the right side indicate number of days from SES implantation to ST. APT = antiplatelet therapy, DAPT = dual-APT, SAPT = single-APT, SES = sirolimus-eluting stents, and ST = stent thrombosis.

In the current analysis, APT status just 1-day before the event might not be causally related to the event, if APT status had changed a few days before the event. Therefore, we evaluated APT status during 30 days before the event in 73 patients with definite ST in the SES stratum (Fig 10). In 2 patients with dual- or single-APT just 1-day before the event, both aspirin and thienopyridines had been discontinued until 3 days before the event. We could not deny the possibility that these ST events were causally related to no-APT, even if these patients were classified as either dual- or single-APT patients according to APT status just 1-day before the event. However, in the remaining 71 patients, same APT status was maintained until the event for at least 1 week. Therefore, APT status just 1-day before the event seemed to correctly reflect APT status during the week before the event in the vast majority of patients with definite ST of SES.

## Discussion

The main findings of the current novel analysis linking serious cardiovascular events after coronary stent implantation to APT status just 1-day before the events are as follows; (1) Discontinuation of both aspirin and thienopyridines was associated with significantly higher risk for ST up to 4-year after SES implantation, while higher risk for ST after discontinuation of both aspirin and thienopyridines was evident only until 6-month after BMS implantation; (2) Discontinuation of both aspirin and thienopyridines was also associated with significantly higher risk for spontaneous MI and stroke; (3) Discontinuation of either thienopyridine or aspirin only as compared with dual-APT was not associated with higher risk for serious cardiovascular events beyond 1-month after coronary stent implantation, except for the marginally higher risk for ST after SES implantation.

Antiplatelet agents consists of several classes including, (1) Cyclooxygenase-1 (COX-1) inhibitors like aspirin, (2) P2Y12 (Adenosine diphosphate [ADP] receptor) inhibitors like thienopyridines represented by Clopidogrel and Ticlopidine, (3) Phosphodiesterase inhibitors like Cilostazol, (4) Glycoprotein IIB/IIIA inhibitors or others. Dual-APT mainly with aspirin and thienopyridines is the cornerstone therapy to prevent ST or other ischemic event within 30 days after coronary stent implantation [25, 26]. However, optimal duration of dual-APT after coronary stent implantation is still a controversial issue because APT discontinuation may increase the risk of ST or adverse event.

Previous reports evaluating the influence of APT discontinuation on ST could be classified into the following 5 types according to their methodologies: (1) randomized controlled trials comparing single-APT with dual-APT at various timings after coronary stent implantation [10–15], (2) landmark analyses based on APT-status at various landmark points after coronary stent implantation [2, 5, 8, 9, 16, 17, 20], (3) analyses using APT discontinuation as a time-updated covariate [1, 3, 4, 6, 18, 19], (4) multivariate adaptive regression splines approach correlating the durations of thienopyridines therapy with occurrence of ST [7], and (5) evaluation of the ST rates according to the presence or absence of APT discontinuation in several time intervals after coronary stent implantation [6, 8, 9]. Each methodology has its own advantages and shortcomings. The randomized controlled trial is the most robust way to define the optimal duration of dual-APT. However, impact of temporary APT discontinuation on cardiovascular outcome could not be assessed by this method. The landmark analysis has the same limitations as the randomized controlled trial together with limitations related to selection bias and unmeasured confounders inherent to observational studies. Analysis using discontinuation as a time-updated covariate could assess temporary APT discontinuation. However, this method could not address the relation between the timing of APT discontinuation after stent implantation and risk for ST. This approach also could not evaluate the APT status at the time of events. Multivariate adaptive regression splines approach correlating the durations of thienopyridines therapy with occurrence of ST is also limited by the failure to evaluate the influences of temporary APT discontinuation. Evaluation of the ST rates according to the presence or absence of APT discontinuation in several time intervals after coronary stent implantation could address the influence of temporary APT discontinuation on ST according to the timing of APT discontinuation. However, this approach also could not evaluate the APT status at the time of events. Finally, many previous studies with a few exceptions [8, 9, 18] did not address the impact of aspirin discontinuation on subsequent ST.

In the current analysis, we linked serious cardiovascular events after coronary stent implantation to APT status just 1-day before the events. The biggest advantage of this methodology was its ability to evaluate the APT status at the time of cardiovascular events. The timing of APT discontinuation after stent implantation was also taken into consideration. Discontinuation of aspirin and thienopyridines was separately analyzed. Furthermore, we evaluated the influence of APT discontinuation on serious cardiovascular events other than ST including spontaneous MI and stroke.

It was noteworthy that nearly half of the SES patients still continued dual-APT even at 5-year after coronary stent implantation. Many of these patients seemed to have continued prolonged dual-APT mainly due to concerns on very late ST of SES, because the rate of dual-APT continuation in the SES stratum remained high even after exclusion of those patients with other indications of prolonged dual-APT such as prior stroke and peripheral vascular disease. In several recent reports with second-generation DES, nearly 90% of patients continued dual-APT at 1-year [27, 28], suggesting that the duration of dual-APT seemed to have been further prolonged despite use of more improved DES. Long-term treatment with dual-APT was associated with concerns on bleeding complications and with profound economic burden. Current ACCF/AHA/SCAI guideline recommends prolonged (for at least 1 year) use of dual-APT after DES implantation [29]. However, in the previous randomized controlled trials and observational studies, prolonged dual-APT as compared with dual-APT regimen shorter than 1-year after DES implantation did not reduce serious cardiovascular events, but increased bleeding events [6–13]. However, the recently reported DAPT trial suggested that prolonged DAPT up to 30 months provided clinical benefits in reducing ST and MI [30]. Therefore, the optimal duration of DAPT after coronary stent implantation is still controversial. In the current analysis, single-APT was not associated with higher risk for serious cardiovascular events even beyond 1-month after stent implantation, except for the marginally higher risk for ST after SES implantation. Therefore, dual-APT shorter than 6-month might be as safe as dual-APT of longer duration in the current clinical practice mainly using second-generation DES, which has been reported to be associated with lower risk for ST than first-generation DES and even BMS [31].

Another important observation in the current study was that discontinuation of both aspirin and thienopyridines was associated with markedly increased risk for ST. The finding was consistent with our previous report from the j-Cypher registry [8]. The current study had much longer duration of follow-up and much greater number of ST events than the previous report, confirming the increased risk for ST with no APT coverage after SES implantation. In the BMS group, increased risk for ST with no APT coverage was only seen within 6-month after PCI. However, discontinuation of both aspirin and thienopyridines was associated with increased risk for spontaneous MI and stroke regardless of the type of stents implanted, which was consistent with the previous reports from a study after coronary stenting [18] and a limited number of secondary prevention studies [32–34]. Therefore, it seems crucially important to notice the danger of no APT coverage to patients, general practitioners, surgeons, dentists, gastrointestinal specialists, and all the medical professions including nurses and pharmacists who take care of patients with coronary artery disease.

This study has several limitations. First, we cannot deny the possibility of recall bias when the information on APT status was obtained from the patients and/or their relatives. Second, we did not assess the actual compliance and adherence to APT. Third, PARIS registry reported that cardiac events after cessation of dual-APT depend on the clinical reasons for cessation and early risk for events due to disruption (cessation of APT due to bleeding or non-compliance) is substantial irrespective of stent type [18]. We did not incorporate the clinical reasons for discontinuation in the current analysis. However, it should be noted that discontinuation of both aspirin and thienopyridines is common in cases of disruption, suggesting that no APT coverage might be more closely related to serious cardiovascular events than disruption per se. Fourth, in patients with changes in status of APT immediately before the event, APT status just 1-day before the event might not causally related to the event. However, this was demonstrated not to be a major issue in the case of ST after SES implantation. Fifth, the number of ST events in each time interval was still too small to conclude how long the increased risk of no APT coverage for ST would persist after SES implantation. However, we had enough number of events in aggregate to argue the increased risk of no APT coverage for serious cardiovascular events including ST, spontaneous MI, and stroke. Sixth, we could not identify the exact date of discontinuation in 8.3% of patients not on the drug at follow-up. However, a sensitivity analysis excluding those patients without date of discontinuation revealed fully consistent results with those from the entire cohort (data not shown). Finally, first-generation SES is no longer used in the clinical arena. However, there are millions of patients who had already received first-generation SES. Optimization of APT after first-generation SES implantation is still crucially important to reduce the late adverse events in these patients. Also, no APT coverage was associated with higher risk for events unrelated to the stent types such as spontaneous MI and stroke. Therefore, the current study results would also be applicable to those patients who had received second generation DES, although the risk for ST would be much different between the first and second generation DES.

## Conclusions

Discontinuation of both aspirin and thienopyridines was associated with increased risk for serious cardiovascular events including ST, spontaneous MI and stroke beyond 1-month after coronary stenting.

## Supporting Information

S1 Appendix List of participating centers and investigators for the CREDO-Kyoto PCI/CABG Registry Cohort-2. (DOCX)

S1 Protocol CREDO-Kyoto PCI/CABG Registry Cohort-2 Study protocol. (DOC)

S1 Table Predictors of Each End-point Event Beyond 30 Days (Multivariable Cox Regression Models). (DOCX)

S1 Fig Prevalence of Each APT Status During Follow-up Censored After Repeated PCI in the SES group (A), and in the BMS group (B). APT = antiplatelet therapy, BMS = bare-metal stents, DAPT = dual-APT, PCI = percutaneous coronary intervention, and SES = sirolimus-eluting stents.
(TIF)

S2 Fig Histograms of the Duration from Drug Cessation to the Onset of Definite ST in the SES Stratum (A), and in the BMS Stratum (B). Values are expressed as median (interquartile range) days. APT = antiplatelet therapy, BMS = bare-metal stents, SAPT = single-APT, SES = sirolimus-eluting stents, and ST = stent thrombosis. As for the definitions of the duration, see Table 3 in the manuscript.
(TIF)

S3 Fig Histograms of the Duration from Drug Cessation to the Onset of Spontaneous Myocardial Infarction in the SES Stratum (A), and in the BMS Stratum (B). Values are expressed as median (interquartile range) days. APT = antiplatelet therapy, BMS = bare-metal stents, MI = myocardial infarction, SAPT = single-APT, and SES = sirolimus-eluting stents. As for the definitions of the duration, see Table 3 in the manuscript.
(TIF)

S4 Fig Histograms of the Duration from Drug Cessation to the Onset of Stroke in the SES Stratum (A), and in the BMS Stratum (B). Values are expressed as median (interquartile range) days. APT = antiplatelet therapy, BMS = bare-metal stents, SAPT = single-APT, and SES = sirolimus-eluting stents. As for the definitions of the duration, see Table 3 in the manuscript.
(TIF)

## References

[1] Iakovou I, Schmidt T, Bonizzoni E, Ge L, Sangiorgi GM, Stankovic G, et al Incidence, predictors, and outcome of thrombosis after successful implantation of drug-eluting stents. JAMA. 2005;293: 2126–2130. 15870416 10.1001/jama.293.17.2126

[2] Eisenstein EL, Anstrom KJ, Kong DF, Shaw LK, Tuttle RH, Mark DB, et al Clopidogrel use and long-term clinical outcomes after drug-eluting stent implantation. JAMA. 2007;297: 159–168. 17148711 10.1001/jama.297.2.joc60179

[3] Park DW, Park SW, Park KH, Lee BK, Kim YH, Lee CW, et al Frequency of and risk factors for stent thrombosis after drug-eluting stent implantation during long-term follow-up. Am J Cardiol. 2006;98: 352–356. 16860022 10.1016/j.amjcard.2006.02.039

[4] Ho PM, Fihn SD, Wang L, Bryson CL, Lowy E, Maynard C, et al Clopidogrel and long-term outcomes after stent implantation for acute coronary syndrome. Am Heart J. 2007;154: 846–851. 17967588 10.1016/j.ahj.2007.08.028

[5] Brar SS, Kim J, Brar SK, Zadegan R, Ree M, Liu IL, et al Long-term outcomes by clopidogrel duration and stent type in a diabetic population with de novo coronary artery lesions. J Am Coll Cardiol. 2008;51: 2220–2227. 10.1016/j.jacc.2008.01.063 18534267

[6] Airoldi F, Colombo A, Morici N, Latib A, Cosgrave J, Buellesfeld L, et al Incidence and predictors of drug-eluting stent thrombosis during and after discontinuation of thienopyridine treatment. Circulation. 2007;116: 745–754. 17664375 10.1161/CIRCULATIONAHA.106.686048

[7] Schulz S, Schuster T, Mehilli J, Byrne RA, Ellert J, Massberg S, et al Stent thrombosis after drug-eluting stent implantation: incidence, timing, and relation to discontinuation of clopidogrel therapy over a 4-year period. Eur Heart J. 2009;30: 2714–2721. 10.1093/eurheartj/ehp275 19596658

[8] Kimura T, Morimoto T, Nakagawa Y, Tamura T, Kadota K, Yasumoto H, et al Antiplatelet therapy and stent thrombosis after sirolimus-eluting stent implantation. Circulation. 2009;119: 987–995. 10.1161/CIRCULATIONAHA.108.808311 19204304

[9] Tada T, Natsuaki M, Morimoto T, Furukawa Y, Nakagawa Y, Byrne RA, et al Duration of dual antiplatelet therapy and long-term clinical outcome after coronary drug-eluting stent implantation: landmark analyses from the CREDO-Kyoto PCI/CABG Registry Cohort-2. Circ Cardiovasc Interv. 2012;5: 381–391. 10.1161/CIRCINTERVENTIONS.111.967463 22619260

[10] Valgimigli M, Campo G, Monti M, Vranckx P, Percoco G, Tumscitz C, et al Short- versus long-term duration of dual-antiplatelet therapy after coronary stenting: a randomized multicenter trial. Circulation. 2012;125: 2015–26. 10.1161/CIRCULATIONAHA.111.071589 22438530

[11] Gwon HC, Hahn JY, Park KW, Song YB, Chae IH, Lim DS, et al Six-month versus 12-month dual antiplatelet therapy after implantation of drug-eluting stents: the Efficacy of Xience/Promus Versus Cypher to Reduce Late Loss After Stenting (EXCELLENT) randomized, multicenter study. Circulation. 2012;125: 505–513. 10.1161/CIRCULATIONAHA.111.059022 22179532

[12] Kim BK, Hong MK, Shin DH, Nam CM, Kim JS, Ko YG, et al A new strategy for discontinuation of dual antiplatelet therapy: the RESET Trial (REal Safety and Efficacy of 3-month dual antiplatelet Therapy following Endeavor zotarolimus-eluting stent implantation). J Am Coll Cardiol. 2012;60: 1340–1348. 10.1016/j.jacc.2012.06.043 22999717

[13] Feres F, Costa RA, Abizaid A, Leon MB, Marin-Neto JA, Botelho RV, et al Three vs twelve months of dual antiplatelet therapy after zotarolimus-eluting stents: the OPTIMIZE randomized trial. JAMA. 2013;310: 2510–2522. 10.1001/jama.2013.282183 24177257

[14] Park SJ, Park DW, Kim YH, Kang SJ, Lee SW, Lee CW, et al Duration of dual antiplatelet therapy after implantation of drug-eluting stents. N Engl J Med. 2010;362: 1374–1382. 10.1056/NEJMoa1001266 20231231

[15] Lee CW, Ahn JM, Park DW, Kang SJ, Lee SW, Kim YH, et al Optimal duration of dual antiplatelet therapy after drug-eluting stent implantation: a randomized, controlled trial. Circulation. 2014;129: 304–312. 10.1161/CIRCULATIONAHA.113.003303 24097439

[16] Stone GW, Ellis SG, Colombo A, Grube E, Dawkins KD, Friedman M, et al Effect of prolonged thienopyridine use after drug-eluting stent implantation (from the TAXUS landmark trials data). Am J Cardiol. 2008;102: 1017–1022. 10.1016/j.amjcard.2008.05.050 18929702

[17] Kimura T, Morimoto T, Nakagawa Y, Kadota K, Nozaki Y, Tada T, et al Antiplatelet therapy and long-term clinical outcome after sirolimus-eluting stent implantation: 5-year outcome of the j-Cypher registry. Cardiovasc Interv Ther. 2012;27: 181–188. 22695921 10.1007/s12928-012-0109-0

[18] Mehran R, Baber U, Steg PG, Ariti C, Weisz G, Witzenbichler B, et al Cessation of dual antiplatelet treatment and cardiac events after percutaneous coronary intervention (PARIS): 2 year results from a prospective observational study. Lancet. 2013;382: 1714–1722. 10.1016/S0140-6736(13)61720-1 24004642

[19] Kedhi E, Stone GW, Kereiakes DJ, Serruys PW, Parise H, Fahy M, et al Stent thrombosis: insights on outcomes, predictors and impact of dual antiplatelet therapy interruption from the SPIRIT II, SPIRIT III, SPIRIT IV and COMPARE trials. EuroIntervention. 2012;8: 599–606. 10.4244/EIJV8I5A92 22995087

[20] Mulukutla SR, Marroquin OC, Vlachos HA, Selzer F, Toma C, Kip KE, et al Benefit of long-term dual anti-platelet therapy in patients treated with drug-eluting stents: from the NHLBI dynamic registry. Am J Cardiol. 2013;111: 486–492. 10.1016/j.amjcard.2012.10.030 23211356 PMC3579582

[21] Kimura T, Morimoto T, Furukawa Y, Nakagawa Y, Kadota K, Iwabuchi M, et al Long-term safety and efficacy of sirolimus-eluting stents versus bare-metal stents in real world clinical practice in Japan. Cardiovasc Interv Ther. 2011;26: 234–245. 10.1007/s12928-011-0065-0 24122590

[22] Natsuaki M, Morimoto T, Furukawa Y, Nakagawa Y, Kadota K, Yamaji K, et al Late Adverse Events After Implantation of Sirolimus-Eluting Stent and Bare-Metal Stent: Long-Term (5–7 Years) Follow-Up of the Coronary Revascularization Demonstrating Outcome Study-Kyoto Registry Cohort-2. Circ Cardiovasc Interv. 2014;7: 168–179 10.1161/CIRCINTERVENTIONS.113.000987 24550439

[23] Mauri L, Hsieh W, Massaro JM, Ho KK, D’Agostino R, Cutlip DE. Stent thrombosis in randomized clinical trials of drug-eluting stents. N Engl J Med. 2007;356: 1020–1029. 17296821 10.1056/NEJMoa067731

[24] Thygesen K, Alpert JS, Jaffe AS, Simoons ML, Chaitman BR, White HD, et al Third universal definition of myocardial infarction. J Am Coll Cardiol. 2012;60: 1581–1598. 10.1016/j.jacc.2012.08.001 22958960

[25] Leon MB, Baim DS, Popma JJ, Gordon PC, Cutlip DE, Ho KK, et al A clinical trial comparing three antithrombotic-drug regimens after coronary-artery stenting. Stent Anticoagulation Restenosis Study Investigators. N Engl J Med 1998;339: 1665–1671. 9834303 10.1056/NEJM199812033392303

[26] Schömig A, Neumann FJ, Kastrati A, Schühlen H, Blasini R, Hadamitzky M, et al A randomized comparison of antiplatelet and anticoagulant therapy after the placement of coronary-artery stents. N Engl J Med. 1996;334: 1084–1089. 8598866 10.1056/NEJM199604253341702

[27] Kimura T, Morimoto T, Natsuaki M, Shiomi H, Igarashi K, Kadota K, et al Comparison of everolimus-eluting and sirolimus-eluting coronary stents: 1-year outcomes from the Randomized Evaluation of Sirolimus-eluting Versus Everolimus-eluting stent Trial (RESET). Circulation. 2012;126: 1225–1236. 22824435 10.1161/CIRCULATIONAHA.112.104059

[28] Natsuaki M, Kozuma K, Morimoto T, Kadota K, Muramatsu T, Nakagawa Y, et al Biodegradable polymer biolimus-eluting stent versus durable polymer everolimus-eluting stent: a randomized, controlled, noninferiority trial. J Am Coll Cardiol. 2013;62: 181–190. 10.1016/j.jacc.2013.04.045 23684673

[29] Levine GN, Bates ER, Blankenship JC, Bailey SR, Bittl JA, Cercek B, et al 2011 ACCF/AHA/SCAI Guideline for Percutaneous Coronary Intervention: a report of the American College of Cardiology Foundation/American Heart Association Task Force on Practice Guidelines and the Society for Cardiovascular Angiography and Interventions. Circulation. 2011;124: e574–e651. 10.1161/CIR.0b013e31823ba622 22064601

[30] Mauri L, Kereiakes DJ, Yeh RW, Driscoll-Shempp P, Cutlip DE, Steg PG, et al Twelve or 30 months of dual antiplatelet therapy after drug-eluting stents. N Engl J Med. 2014;371: 2155–66. 10.1056/NEJMoa1409312 25399658 PMC4481318

[31] Palmerini T, Biondi-Zoccai G, Della Riva D, Stettler C, Sangiorgi D, D'Ascenzo F, et al Stent thrombosis with drug-eluting and bare-metal stents: evidence from a comprehensive network meta-analysis. Lancet. 2012;379: 1393–1402. 10.1016/S0140-6736(12)60324-9 22445239

[32] Rodríguez LAG, Cea-Soriano L, Martín-Merino E, Johansson S. Discontinuation of low dose aspirin and risk of myocardial infarction: case-control study in UK primary care. BMJ. 2011;343: d4094 10.1136/bmj.d4094 21771831 PMC3139911

[33] García Rodríguez LA, Cea Soriano L, Hill C, Johansson S. Increased risk of stroke after discontinuation of acetylsalicylic acid: a UK primary care study. Neurology. 2011;76: 740–746. 10.1212/WNL.0b013e31820d62b5 21270415

[34] Weimar C, Cotton D, Sha N, Sacco RL, Bath PM, Weber R, et al Discontinuation of antiplatelet study medication and risk of recurrent stroke and cardiovascular events: results from the PRoFESS study. Cerebrovasc Dis. 2013;35: 538–543. 10.1159/000351144 23816610

