# Supplementary material for: Antiplatelet Therapy Discontinuation and the Risk of Serious Cardiovascular Events after Coronary Stenting: Observations from the CREDO-Kyoto Registry Cohort-2
Source: PLoS One. 2015 Apr 8;10(4):e0124314. doi: 10.1371/journal.pone.0124314 (PMC4390156; doi:10.1371/journal.pone.0124314)
Supplement: S1 Appendix — (DOCX) [file pone.0124314.s001.docx]

**S1 Appendix. List of participating centers and investigators for the CREDO-Kyoto PCI/CABG Registry Cohort-2**

**Cardiology**

Kyoto University Hospital: Takeshi Kimura

Kishiwada City Hospital: Mitsuo Matsuda, Hirokazu Mitsuoka

Tenri Hospital: Yoshihisa Nakagawa

Hyogo Prefectural Amagasaki Hospital: Hisayoshi Fujiwara, Yoshiki Takatsu, Ryoji Taniguchi

Kitano Hospital: Ryuji Nohara

Koto Memorial Hospital: Tomoyuki Murakami, Teruki Takeda

Kokura Memorial Hospital: Masakiyo Nobuyoshi, Masashi Iwabuchi

Maizuru Kyosai Hospital: Ryozo Tatami

Nara Hospital, Kinki University Faculty of Medicine: Manabu Shirotani

Kobe City Medical Center General Hospital: Toru Kita, Yutaka Furukawa, Natsuhiko Ehara

Nishi-Kobe Medical Center: Hiroshi Kato, Hiroshi Eizawa

Kansai Denryoku Hospital: Katsuhisa Ishii

Osaka Red Cross Hospital: Masaru Tanaka

University of Fukui Hospital: Jong-Dae Lee, Akira Nakano

Shizuoka City Shizuoka Hospital: Akinori Takizawa

Hamamatsu Rosai Hospital: Masaaki Takahashi

Shiga University of Medical Science Hospital: Minoru Horie, Hiroyuki Takashima

Japanese Red Cross Wakayama Medical Center: Takashi Tamura

Shimabara Hospital: Mamoru Takahashi

Kagoshima University Medical and Dental Hospital: Chuwa Tei, Shuichi Hamasaki

Shizuoka General Hospital: Hirofumi Kambara, Osamu Doi, Satoshi Kaburagi

Kurashiki Central Hospital: Kazuaki Mitsudo, Kazushige Kadota

Mitsubishi Kyoto Hospital: Shinji Miki, Tetsu Mizoguchi

Kumamoto University Hospital: Hisao Ogawa, Seigo Sugiyama

Shimada Municipal Hospital: Ryuichi Hattori, Takeshi Aoyama, Makoto Araki

Juntendo University Shizuoka Hospital: Satoru Suwa

**Cardiovascular Surgery**

Kyoto University Hospital: Ryuzo Sakata, Tadashi Ikeda, Akira Marui

Kishiwada City Hospital: Masahiko Onoe

Tenri Hospital: Kazuo Yamanaka

Hyogo Prefectural Amagasaki Hospital: Keiichi Fujiwara, Nobuhisa Ohno

Kokura Memorial Hospital: Michiya Hanyu

Maizuru Kyosai Hospital: Tsutomu Matsushita

Nara Hospital, Kinki University Faculty of Medicine: Noboru Nishiwaki, Yuichi Yoshida

Kobe City Medical Center General Hospital: Yukikatsu Okada, Michihiro Nasu

Osaka Red Cross Hospital: Shogo Nakayama

University of Fukui Hospital: Kuniyoshi Tanaka, Takaaki Koshiji, Koichi Morioka

Shizuoka City Shizuoka Hospital: Mitsuomi Shimamoto, Fumio Yamazaki

Hamamatsu Rosai Hospital: Junichiro Nishizawa

Japanese Red Cross Wakayama Medical Center: Masaki Aota

Shimabara Hospital: Takafumi Tabata

Kagoshima University Medical and Dental Hospital: Yutaka Imoto, Hiroyuki Yamamoto

Shizuoka General Hospital: Katsuhiko Matsuda, Masafumi Nara

Kurashiki Central Hospital: Tatsuhiko Komiya

Mitsubishi Kyoto Hospital: Hiroyuki Nakajima

Kumamoto University Hospital: Michio Kawasuji, Syuji Moriyama

Juntendo University Shizuoka Hospital: Keiichi Tanbara

**Research Institute for Production Development**

Kumiko Kitagawa, Misato Yamauchi, Naoko Okamoto, Yumika Fujino, Saori Tezuka, Asuka Saeki, Miya Hanazawa, Yuki Sato, Chikako Hibi, Hitomi Sasae, Emi Takinami, Yuriko Uchida, Yuko Yamamoto, Satoko Nishida, Mai Yoshimoto, Sachiko Maeda, Izumi Miki, Saeko Minematsu.

**List of clinical event committee members**

Mitsuru Abe (Kyoto Medical Center), Hiroki Shiomi (Kyoto University Hospital), Tomohisa Tada (Kyoto University Hospital), Junichi Tazaki (Kyoto University Hospital), Yoshihiro Kato (Kyoto University Hospital), Mamoru Hayano (Kyoto University Hospital), Akihiro Tokushige (Kyoto University Hospital), Masahiro Natsuaki (Kyoto University Hospital), Tetsu Nakajima (Kyoto University Hospital).
