# Supplementary material for: Antiplatelet Therapy Discontinuation and the Risk of Serious Cardiovascular Events after Coronary Stenting: Observations from the CREDO-Kyoto Registry Cohort-2
Source: PLoS One. 2015 Apr 8;10(4):e0124314. doi: 10.1371/journal.pone.0124314 (PMC4390156; doi:10.1371/journal.pone.0124314)
Supplement: S1 Protocol — (DOC) [file pone.0124314.s002.doc]

**S1 Protocol. CREDO-Kyoto PCI/CABG Registry Cohort-2 Study protocol**

The Coronary REvascularization Demonstrating Outcome study in Kyoto (CREDO-Kyoto) percutaneous coronary intervention (PCI)/coronary artery bypass grafting (CABG) registry cohort-2

2004.4.10 1^st^ version

2004.6.2 revised

2004.9.15 revised

2004.11.3 revised

2006.12.25 revised

2007.1.4 revised　　　　　　　　　　　　　　　 2007.6.29 revised　　　　　　　　　　　　　　　　　　　　　　　　　　　　　　 2007.7.30 revised

**Table of Contents**

0. Schema ・・・・・・・・・・・・2

1. Objectives ・・・・・・・・・・・・3

2. Background and Rationale ・・・・・・・・・・・・3

3. Inclusion Criteria ・・・・・・・・・・・・4

4. Study Method ・・・・・・・・・・・・4

5. Observation Items at Baseline ・・・・・・・・・・・・5

1. Endpoints ・・・・・・・・・・・・5

7. Statistical Considerations ・・・・・・・・・・・・6

8. Ethical Issues ・・・・・・・・・・・・6

9. Study Organization ・・・・・・・・・・・・6

1. Funding Source ・・・・・・・・・・・・9

11. Authorship ・・・・・・・・・・・・9

12. References ・・・・・・・・・・・・9

**0. Schema**

**Study Patients**

Consecutive patients undergoing first coronary revascularization

either with PCI or with CABG from January 2005 to December 2007

(CREDO-Kyoto PCI/CABG Registry Cohort-2)

Baseline Clinical, Angiographic, and Procedural Data

Entry by Clinical Research Coordinators

Follow-up Data (2-4 year)

Entry by Clinical Research Coordinators

Follow-up Data (4-7 year)

Entry by Clinical Research Coordinators

Final Analysis

1. **Objectives**

The current retrospective multicenter observational registry enrolls consecutive patients undergoing first coronary revascularization either with PCI or with CABG from January 2005 to December 2007. The outcome measures evaluated include clinical events such as death, myocardial infarction, stent thrombosis, stroke, hospitalization for worsening heart failure, bleeding, target-lesion revascularization, and any coronary revascularization. We seek to evaluate the practice patterns, long-term clinical outcome, and factors influencing on the outcome after coronary revascularization.

The primary analyses include comparison between PCI and CABG, and comparison between sirolimus-eluting stent (SES) and bare-metal stent (BMS).

As a historical comparison, the practice patterns and outcome in the current registry will be compared with the CREDO-Kyoto cohort-1 that enrolled consecutive patients undergoing first coronary revascularization either with PCI or with CABG from January 2000 to December 2002.

The current study will be conducted to build a basis of scientific evaluation for mal-function of medical devises based on the mid-term plan of Pharmaceuticals and Medical Devices Agency (PMDA) in Japan.

1. **Background and Rationale**

The prevalence of coronary artery disease is increasing due to aging society and westernized dietary habit. Life-style modification and optimal medical therapy are the mainstay in the prevention and treatment of coronary artery disease. However, coronary revascularization either with PCI or CABG has been widely utilized in clinical practice based on the proven benefit of improvement of quality of life by alleviating myocardial ischemia. CABG has been reported to have benefit in improving long-term survival in patients with severe coronary artery disease such as patients with left main coronary artery disease, triple vessel disease, and impaired left ventricular function, while there has been no previous report suggesting improvement of survival by PCI in patients with chronic coronary artery disease. (1-3) Furthermore, in patients with treated diabetes, long-term survival was better with CABG as compared with PCI. (4) However, these results were mainly reported from US and European countries. However, it is not appropriate to apply these study results directly to the clinical practice in Japan with different ethnicity and dietary habit. Cardiovascular event risk in Japanese patients is lower than that in Western patients. An intervention with proven efficacy in high-risk population might not always effective in low risk population such as Japanese.

We previously conducted the CREDO-Kyoto registry cohort-1 that enrolled consecutive patients undergoing first coronary revascularization either with PCI or with CABG from January 2000 to December 2002. This registry was the first large-scale registry comparing PCI with CABG. In March 2004, the first drug-eluting stent, SES, was approved in Japan. SES has been proven to prevent restenosis dramatically in randomized controlled trials conducted outside Japan. (5-7) The current CREDO-Kyoto registry cohort-2 was designed to enroll consecutive patients undergoing first coronary revascularization either with PCI or with CABG after approval of SES in Japan. The current study will shed light on the changes in clinical practices and outcomes of coronary revascularization after introduction of drug eluting stent.

**3. Inclusion Criteria**

Consecutive patients undergoing first coronary revascularization either with PCI or with CABG from January 2005 to December 2007 among 26 participating centers.

1. **Study Method**

Demographic, angiographic, and procedural data were collected from hospital charts or hospital databases according to pre-specified definitions by independent experienced clinical research coordinators from the study management center. Baseline data forms were to be double-checked by a second clinical research coordinator in 10-15% of patients. In the data cleaning process, logical inconsistencies were resolved by audits against the original source documents. Follow-up data were obtained from hospital charts or by contacting patients or referring physicians. Scheduled staged PCI procedures during the index hospitalization or performed within 3 months of the initial procedure were not regarded as follow-up events, but were included in the index procedure. Events such as cardiac death, sudden death, non-cardiac death, myocardial infarction, stent thrombosis, stroke and bleeding were adjudicated by a clinical event committee. Status of antiplatelet therapy during follow-up was also evaluated. Dates of discontinuation of aspirin and thienopyridine were reported separately in the follow-up forms. If either aspirin or thienopyridine was restarted after discontinuation, the dates of restart were also recorded.

**5. Observation Items at Baseline**

5-1. Baseline clinical characteristics

5-2. Angiographic characteristics

5-3. Procedural characteristics

5-4. Discharge Medications

**6. Endpoints**

**6-1. Death**: Death was regarded as cardiac in origin unless obvious non-cardiac causes could be identified. Any death during the index hospitalization was regarded as cardiac death. Sudden death was defined as unexplained death in previously stable patients.

**6-2. Myocardial Infarction**: Myocardial infarction was defined according to the definition in the Arterial Revascularization Therapy Study. Within 1 week of the index procedure, only Q-wave myocardial infarction was adjudicated as myocardial infarction. Spontaneous MI was defined as type 1 in the universal classification.

**6-3. Stent thrombosis**: Stent thrombosis was defined according to the Academic Research Consortium definition.

**6-4. Stroke**: Stroke was defined as ischemic or hemorrhagic stroke either occurring in-hospital or during follow-up requiring hospitalization with symptoms lasting >24 hour.

**6-5. Hospitalization for heart failure**: Hospitalization for heart failure was defined as hospitalization due to worsening heart failure requiring intravenous drug therapy.

**6-6. Bleeding**: Bleeding was defined according to the Global Utilization of Streptokinase and Tissue plasminogen activator for Occluded coronary arteries (GUSTO) classification. GUSTO moderate or severe bleeding was adjudicated as a bleeding event. Procedure-related bleeding was defined as that occurred during hospitalization for percutaneous interventional procedures or surgical procedures.

**6-7. Target-lesion revascularization**: Target-lesion revascularization was defined as either percutaneous coronary intervention or coronary artery bypass grafting due to restenosis or thrombosis of the target lesion that included the proximal and distal edge segments as well as the ostium of the side branches.

**6-8. Any coronary revascularization**: Any coronary revascularization was defined as any PCI or CABG during follow-up excluding scheduled staged PCI performed within 3 months of the index PCI procedure.

**7. Statistical Considerations**

Cumulative incidence is estimated by the Kaplan-Meier method and differences are assessed with the log-rank test. We use Cox proportional hazard models for adjusted analysis with 39 clinically relevant factors as potential independent risk-adjusting variables. The continuous variables are dichotomized by clinically meaningful reference values or median values. Proportional hazard assumptions for potential independent risk-adjusting variables are to be assessed on the plots of log (time) versus log [-log (survival)] stratified by the variable. Center is included in the Cox proportional hazard models as the stratification variables.

**8. Ethical Issues**

The relevant review boards or ethics committees in all 26 participating centers should approve the research protocol. Because of retrospective enrollment, written informed consents from the patients are waived; however, we exclude those patients who refuse participation in the study when contacted for follow-up. This strategy is concordant with the guidelines for epidemiological studies issued by the Ministry of Health, Labor and Welfare of Japan.

**9. Study Organization**

**Principal Investigator**: Takeshi Kimura

Department of Cardiovascular Medicine, Kyoto University Graduate School of Medicine

**Co-Principal Investigator**: Yutaka Furukawa

Department of Cardiovascular Medicine, Kyoto University Graduate School of Medicine

**Study Secretariat**: Research Institute for Production Development

Cardiovascular Clinical Research Promotion Department

Shimogamo-morimoto-cho 15, Sakyo-ward, Kyoto-city, 606-0805

Phone: 075-781-1107 Fax: 075-791-7659

Person in charge of the study: Misato Yamauchi

Person in charge of contract: Kumiko Kitagawa

**Data center**: Department of Cardiovascular Medicine, Graduate School of Medicine, Kyoto

University

Shogoin Kawahara-cho 54, Sakyo-ward, Kyoto-city, 606-8507

Phone: 075-751-4255 Fax: 075-751-3299

Responsible person: Takeshi Kimura

**Statistical analysis**: Takeshi Morimoto

Center for Medical Education, Kyoto University

**Clinical event committee**: Mitsuru Abe (Kyoto Medical Center), Hiroki Shiomi (Kyoto University Hospital), Tomohisa Tada (Kyoto University Hospital), Junichi Tazaki (Kyoto University Hospital), Yoshihiro Kato (Kyoto University Hospital), Mamoru Hayano (Kyoto University Hospital), Akihisa Tokushige (Kyoto University Hospital), Masahiro Natsuaki (Kyoto University Hospital), Tetsu Nakajima (Kyoto University Hospital).

**Participating centers and investigators**:

**Cardiology**

Kyoto University Hospital: Takeshi Kimura

Kishiwada City Hospital: Mitsuo Matsuda, Hirokazu Mitsuoka

Tenri Hospital: Yoshihisa Nakagawa

Hyogo Prefectural Amagasaki Hospital: Hisayoshi Fujiwara, Yoshiki Takatsu, Ryoji Taniguchi

Kitano Hospital: Ryuji Nohara

Koto Memorial Hospital: Tomoyuki Murakami, Teruki Takeda

Kokura Memorial Hospital: Masakiyo Nobuyoshi, Masashi Iwabuchi

Maizuru Kyosai Hospital: Ryozo Tatami

Nara Hospital, Kinki University Faculty of Medicine: Manabu　Shirotani

Kobe City Medical Center General Hospital: Toru Kita, Yutaka Furukawa, Natsuhiko Ehara

Nishi-Kobe Medical Center: Hiroshi Kato, Hiroshi Eizawa

Kansai Denryoku Hospital: Katsuhisa Ishii

Osaka Red Cross Hospital: Masaru Tanaka

University of Fukui Hospital: Jong-Dae Lee, Akira Nakano

Shizuoka City Shizuoka Hospital: Akinori Takizawa

Hamamatsu Rosai Hospital: Masaaki Takahashi

Shiga University of Medical Science Hospital: Minoru Horie, Hiroyuki Takashima

Japanese Red Cross Wakayama Medical Center: Takashi Tamura

Shimabara Hospital: Mamoru Takahashi

Kagoshima University Medical and Dental Hospital: Chuwa Tei, Shuichi Hamasaki

Shizuoka General Hospital: Hirofumi Kambara, Osamu Doi, Satoshi Kaburagi

Kurashiki Central Hospital: Kazuaki Mitsudo, Kazushige Kadota

Mitsubishi Kyoto Hospital: Shinji Miki, Tetsu Mizoguchi

Kumamoto University Hospital: Hisao Ogawa, Seigo Sugiyama

Shimada Municipal Hospital: Ryuichi Hattori, Takeshi Aoyama, Makoto Araki

Juntendo University Shizuoka Hospital: Satoru Suwa

**Cardiovascular Surgery**

Kyoto University Hospital: Ryuzo Sakata, Tadashi Ikeda, Akira Marui

Kishiwada City Hospital: Masahiko Onoe

Tenri Hospital: Kazuo Yamanaka

Hyogo Prefectural Amagasaki Hospital: Keiichi Fujiwara, Nobuhisa Ohno

Kokura Memorial Hospital: Michiya Hanyu

Maizuru Kyosai Hospital: Tsutomu Matsushita

Nara Hospital, Kinki University Faculty of Medicine: Noboru Nishiwaki, Yuichi Yoshida

Kobe City Medical Center General Hospital: Yukikatsu　Okada, Michihiro Nasu

Osaka Red Cross Hospital: Shogo Nakayama

University of Fukui Hospital: Kuniyoshi Tanaka, Takaaki Koshiji, Koichi Morioka

Shizuoka City Shizuoka Hospital: Mitsuomi Shimamoto, Fumio Yamazaki

Hamamatsu Rosai Hospital: Junichiro Nishizawa

Japanese Red Cross Wakayama Medical Center: Masaki Aota

Shimabara Hospital: Takafumi Tabata

Kagoshima University Medical and Dental Hospital: Yutaka Imoto, Hiroyuki Yamamoto

Shizuoka General Hospital: Katsuhiko Matsuda, Masafumi Nara

Kurashiki Central Hospital: Tatsuhiko Komiya

Mitsubishi Kyoto Hospital: Hiroyuki Nakajima

Kumamoto University Hospital: Michio Kawasuji, Syuji Moriyama

Juntendo University Shizuoka Hospital: Keiichi Tanbara

**10. Funding Source**

Pharmaceuticals and Medical Devices Agency (PMDA) in Japan

**11. Authorship**

Authorship for publications from the current study will be decided by the discussion among the principal investigator, co-investigators, and the sponsor.

**12. References**

1. Passamani E, Davis KB, Gillespie MJ, Killip T. A randomized trial of coronary artery bypass surgery. N Eng J Med 1985;312:1665-71.

2. Coronary angioplasty versus medical therapy for angina: the second Randomised Intervention Treatment of Angina (RITA-2) trial. RITA-2 trial participants. Lancet. 1997;350:461-8.

3. Pitt B, Waters D, Brown WV, van Boven AJ, Schwartz L, Title LM, Eisenberg D, Shurzinske L, McCormick LS. Aggressive lipid-lowering therapy compared with angioplasty in stable coronary artery disease. Atorvastatin versus Revascularization Treatment Investigators. N Engl J Med. 1999;341:70-6.

4. Seven-year outcome in the Bypass Angioplasty Revascularization Investigation (BARI) by treatment and diabetic status. J Am Coll Cardiol. 2000;35:1122-9.

5. Morice MC, Serruys PW, Sousa JE, Fajadet J, Ban Hayashi E, Perin M, Colombo A, Schuler G, Barragan P, Guagliumi G, Molnar F, Falotico R; RAVEL Study Group. Randomized Study with the Sirolimus-Coated Bx Velocity Balloon-Expandable Stent in the Treatment of Patients with de Novo Native Coronary Artery Lesions: A randomized comparison of a sirolimus-eluting stent with a standard stent for coronary revascularization. N Engl J Med. 2002;346:1773-80.

6. Moses JW, Leon MB, Popma JJ, Fitzgerald PJ, Holmes DR, O'Shaughnessy C, Caputo RP, Kereiakes DJ, Williams DO, Teirstein PS, Jaeger JL, Kuntz RE; SIRIUS Investigators. Sirolimus-eluting stents versus standard stents in patients with stenosis in a native coronary artery. N Engl J Med. 2003;349:1315-23.

7. Holmes DR Jr, Leon MB, Moses JW, Popma JJ, Cutlip D, Fitzgerald PJ, Brown C, Fischell T, Wong SC, Midei M, Snead D, Kuntz RE. Analysis of 1-year clinical outcomes in the SIRIUS trial: a randomized trial of a sirolimus-eluting stent versus a standard stent in patients at high risk for coronary restenosis. Circulation. 2004;109(5):634-40
