# Supplementary material for: Antiplatelet Therapy Discontinuation and the Risk of Serious Cardiovascular Events after Coronary Stenting: Observations from the CREDO-Kyoto Registry Cohort-2
Source: PLoS One. 2015 Apr 8;10(4):e0124314. doi: 10.1371/journal.pone.0124314 (PMC4390156; doi:10.1371/journal.pone.0124314)
Supplement: S1 Table — (DOCX) [file pone.0124314.s003.docx]

**S1 Table. Predictors of Each End-point Event Beyond 30 Days (Multivariable Cox Regression Models)**

1. **Stent thrombosis**

|  | HR (95%CI) | P Value |
| --- | --- | --- |
| SES (versus BMS) | 1.21 (0.77-1.89) | 0.41 |
| Age >=75 years^*^ | 0.98 (0.60-1.56) | 0.95 |
| Male^*^ | 1.18 (0.73-1.98) | 0.52 |
| Acute myocardial infarction | 1.50 (0.93-2.38) | 0.09 |
| Hypertension | 0.99 (0.61-1.67) | 0.95 |
| Diabetes mellitus with insulin therapy^*^ | 1.08 (0.47-2.16) | 0.84 |
| Current smoking | 1.44 (0.94-2.19) | 0.09 |
| Heart failure | 0.73 (0.39-1.28) | 0.29 |
| Multivessel disease^*^ | 0.78 (0.50-1.19) | 0.25 |
| Previous myocardial infarction | 0.98 (0.47-1.84) | 0.94 |
| Dialysis^*^ | 1.61 (0.48-4.00) | 0.40 |
| Atrial fibrillation^*^ | 1.32 (0.61-2.50) | 0.46 |
| Target of proximal LAD^*^ | 2.05 (1.31-3.27) | 0.001 |
| Target of Unprotected LMCA | 1.69 (0.51-4.20) | 0.35 |
| Target of CTO | 1.76 (0.97-3.03) | 0.06 |
| Target of bifurcation | 1.07 (0.69-1.65) | 0.75 |
| Side-branch stenting | 0.69 (0.20-1.77) | 0.47 |
| Total stent length >28mm | 1.67 (1.05-2.64) | .03 |
| Minimum stent size <3.0mm | 1.15 (0.74-1.78) | 0.54 |

1. **Spontaneous myocardial infarction**

|  | HR (95%CI) | P Value |
| --- | --- | --- |
| SES (versus BMS) | 0.63 (0.46-0.86) | 0.004 |
| Age >=75 years^*^ | 1.51 (1.10-2.06) | 0.01 |
| Male^*^ | 1.33 (0.95-1.88) | 0.10 |
| BMI <25 | 0.90 (0.68-1.22) | 0.51 |
| Acute myocardial infarction | 1.42 (0.99-2.04) | 0.06 |
| Hypertension | 0.86 (0.58-1.29) | 0.45 |
| Diabetes mellitus with insulin therapy^*^ | 1.15 (0.68-1.83) | 0.59 |
| Current smoking | 1.21 (0.89-1.63) | 0.23 |
| Heart failure | 1.20 (0.81-1.75) | 0.36 |
| Shock at presentation | 0.45 (0.18-0.97) | 0.04 |
| Multivessel disease^*^ | 1.57 (1.16-2.12) | 0.003 |
| Mitral regurgitation grade 3/4 | 0.86 (0.38-1.67) | 0.67 |
| Previous myocardial infarction | 1.35 (0.86-2.05) | 0.19 |
| Previous stroke^*^ | 0.85 (0.51-1.33) | 0.49 |
| Peripheral vascular disease | 1.07 (0.63-1.73) | 0.80 |
| eGFR <30 ml/min/1,73m^2^, not on dialysis^*^ | 2.50 (1.40-4.22) | 0.003 |
| Dialysis^*^ | 3.43 (1.82-6.09) | <0.001 |
| Atrial fibrillation^*^ | 1.32 (0.78-2.13) | 0.29 |
| Anemia (Hb <11 g/dl) | 1.10 (0.69-1.70) | 0.70 |
| Platelet <100 10^9^/L | 0.33 (0.02-1.48) | 0.18 |
| COPD | 1.40 (0.69-2.53) | 0.33 |
| Liver cirrhosis | 1.71 (0.84-3.10) | 0.13 |
| Malignancy | 1.01 (0.61-1.59) | 0.97 |
| Target or proximal LAD^*^ | 0.93 (0.70-1.23) | 0.60 |
| Target of Unprotected LMCA | 1.57 (0.69-3.12) | 0.26 |
| Target of CTO | 0.70 (0.40-1.16) | 0.18 |
| Target of bifurcation | 0.89 (0.63-1.24) | 0.49 |
| Side-branch stenting | 0.79 (0.30-1.74) | 0.58 |
| Total stent length >28mm | 1.02 (0.75-1.39) | 0.89 |
| Minimum stent size <3.0mm | 1.15 (0.85-1.56) | 0.36 |
| Cilostazol | 0.68 (0.45-1.00) | 0.05 |
| Statins | 0.90 (0.68-1.19) | 0.46 |
| Beta-blocker | 1.51 (1.13-2.03) | 0.006 |
| ACE-I/ARB | 0.85 (0.63-1.16) | 0.31 |
| Nitrates | 0.95 (0.71-1.27) | 0.73 |
| Calcium channel blockers | 1.08 (0.80-1.46) | 0.61 |
| Nicorandil | 1.10 (0.81-1.49) | 0.54 |
| Warfarin | 0.92 (0.52-1.55) | 0.77 |
| Proton pump inhibitors | 1.17 (0.84-1.62) | 0.35 |
| H2-blockers | 0.84 (0.59-1.18) | 0.32 |

1. **Stroke**

|  | HR (95%CI) | P Value |
| --- | --- | --- |
| SES (versus BMS) | 1.11 (0.92-1.34) | 0.29 |
| Age >=75 years^*^ | 1.74 (1.44-2.09) | <0.001 |
| Male^*^ | 1.18 (0.96-1.45) | 0.11 |
| BMI<25 | 1.19 (0.98-1.45) | 0.08 |
| Acute myocardial infarction | 0.88 (0.69-1.11) | 0.28 |
| Hypertension | 1.12 (0.86-1.46) | 0.41 |
| Diabetes mellitus with insulin therapy^*^ | 1.30 (0.97-1.72) | 0.08 |
| Current smoking | 0.99 (0.81-1.21) | 0.94 |
| Heart failure | 1.03 (0.81-1.31) | 0.79 |
| Shock at presentation | 0.95 (0.56-1.55) | 0.84 |
| Multivessel disease^*^ | 1.23 (1.03-1.48) | 0.03 |
| Mitral regurgitation grade 3/4 | 0.90 (0.58-1.35) | 0.63 |
| Previous myocardial infarction | 1.00 (0.76-1.31) | 0.97 |
| Previous stroke^*^ | 2.10 (1.70-2.58) | <0.001 |
| Peripheral vascular disease | 1.42 (1.09-1.83) | 0.009 |
| eGFR <30 ml/min/1,73m^2^, not on dialysis^*^ | 1.44 (0.97-2.06) | 0.07 |
| Dialysis^*^ | 2.08 (1.42-2.99) | <0.001 |
| Atrial fibrillation^*^ | 1.95 (1.49-2.53) | <0.001 |
| Anemia (Hb <11 g/dl) | 1.37 (1.06-1.76) | 0.02 |
| Platelet <100 10^9^/L | 1.01 (0.46-1.90) | 0.98 |
| COPD | 1.25 (0.81-1.83) | 0.30 |
| Liver cirrhosis | 0.86 (0.49-1.39) | 0.56 |
| Malignancy | 0.95 (0.70-1.25) | 0.71 |
| Target or proximal LAD^*^ | 0.95 (0.80-1.14) | 0.60 |
| Target of Unprotected LMCA | 1.32 (0.82-2.02) | 0.24 |
| Target of CTO | 1.11 (0.84-1.45) | 0.46 |
| Target of bifurcation | 1.08 (0.88-1.31) | 0.48 |
| Side-branch stenting | 1.09 (0.69-1.67) | 0.70 |
| Total stent length >28mm | 0.89 (0.74-1.09) | 0.26 |
| Minimum stent size <3.0mm | 1.07 (0.89-1.29) | 0.45 |
| Cilostazol | 1.03 (0.82-1.29) | 0.79 |
| Statins | 0.84 (0.71-1.01) | 0.06 |
| Beta-blocker | 0.96 (0.79-1.15) | 0.64 |
| ACE-I/ARB | 1.10 (0.92-1.33) | 0.30 |
| Nitrates | 0.90 (0.75-1.07) | 0.25 |
| Calcium channel blockers | 0.96 (0.80-1.15) | 0.66 |
| Nicorandil | 0.93 (0.76-1.14) | 0.51 |
| Warfarin | 1.12 (0.83-1.49) | 0.46 |
| Proton pump inhibitors | 1.02 (0.83-1.27) | 0.82 |
| H2-blockers | 1.13 (0.92-1.38) | 0.23 |

*: Variables considered in multivariable logistic regression model in Table 4. Diabetes was used as a whole, not limited to insulin users and chronic kidney disease was used as one variable including both dialysis patients and no-dialysis patients.

ACE-I=angiotensin converting enzyme inhibitors, ARB=angiotensin II receptor blockers, BMI=body mass index, BMS=bare-metal stents, COPD=chronic obstructive pulmonary disease, CTO=chronic total occlusion, eGFR=estimated glomerular filtration rate, LAD=left anterior descending coronary artery, LMCA=left main coronary artery, and SES=sirolimus-eluting stents.
