# Supplementary figures and images for: Antiplatelet Therapy Discontinuation and the Risk of Serious Cardiovascular Events after Coronary Stenting: Observations from the CREDO-Kyoto Registry Cohort-2
Source: PLoS One. 2015 Apr 8;10(4):e0124314. doi: 10.1371/journal.pone.0124314 (PMC4390156; doi:10.1371/journal.pone.0124314)

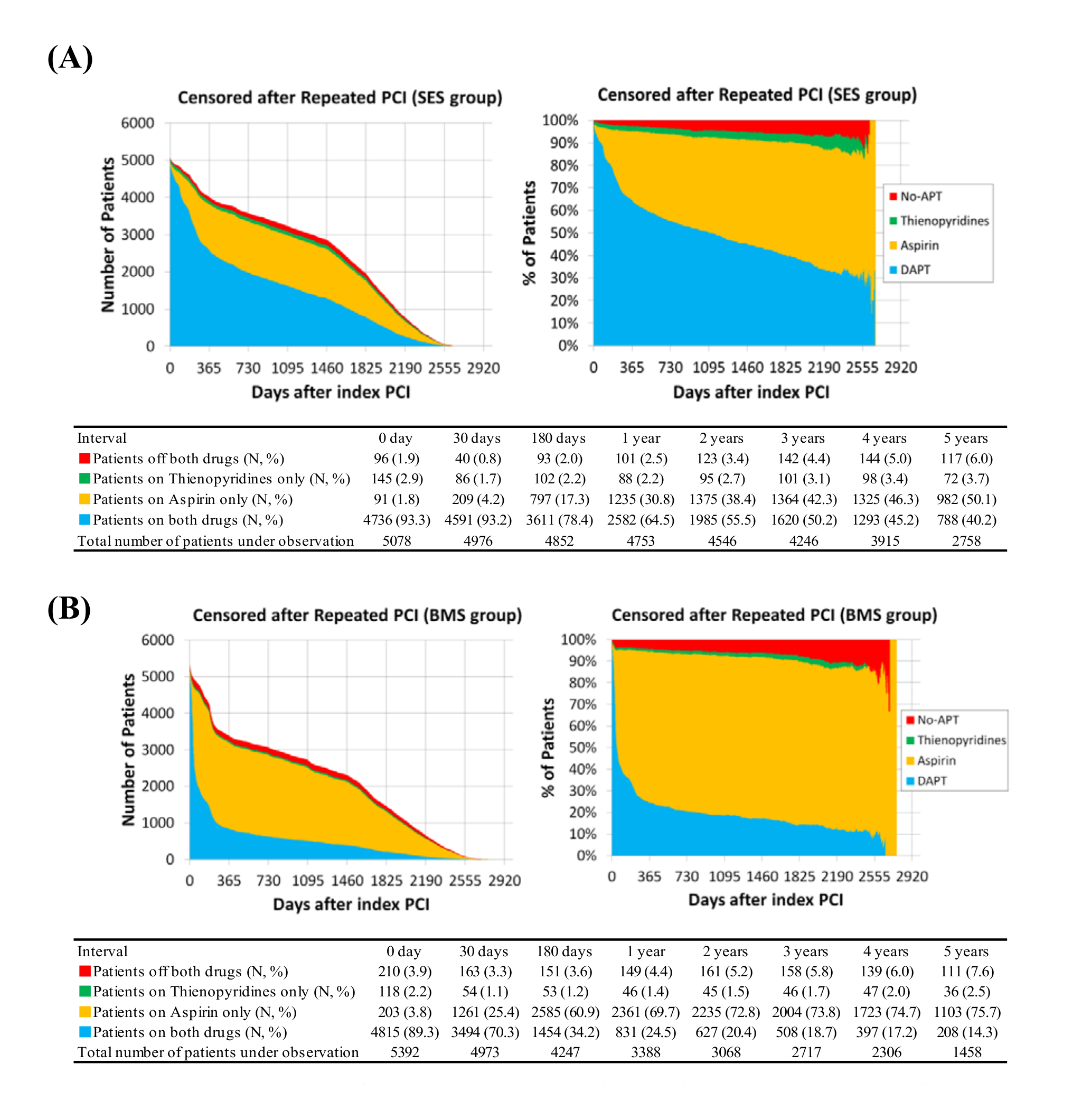

Supplement: S1 Fig — APT = antiplatelet therapy, BMS = bare-metal stents, DAPT = dual-APT, PCI = percutaneous coronary intervention, and SES = sirolimus-eluting stents. (TIF) [file pone.0124314.s004.tif]

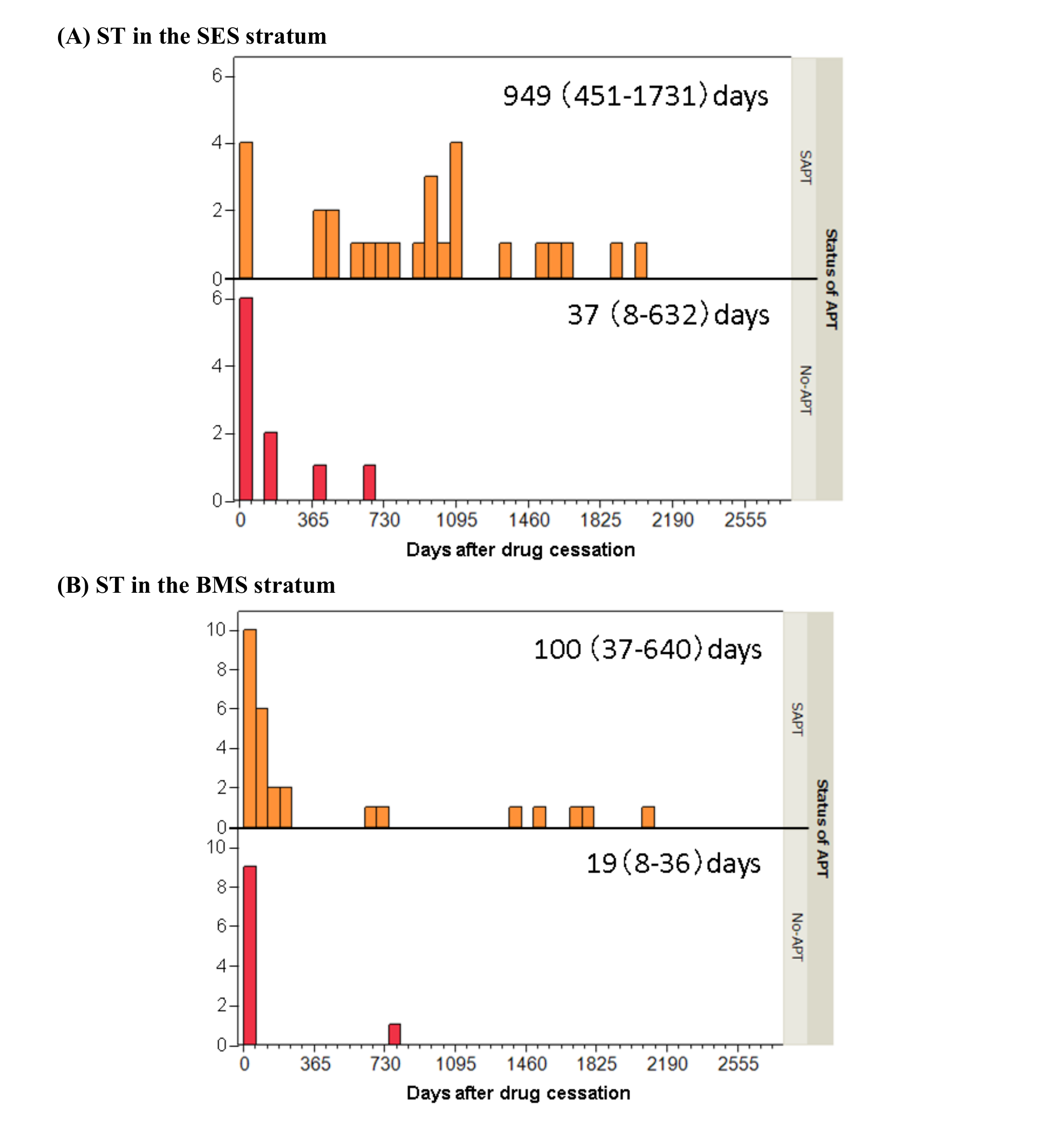

Supplement: S2 Fig — Values are expressed as median (interquartile range) days. APT = antiplatelet therapy, BMS = bare-metal stents, SAPT = single-APT, SES = sirolimus-eluting stents, and ST = stent thrombosis. As for the definitions of the duration, see Table 3 in the manuscript. (TIF) [file pone.0124314.s005.tif]

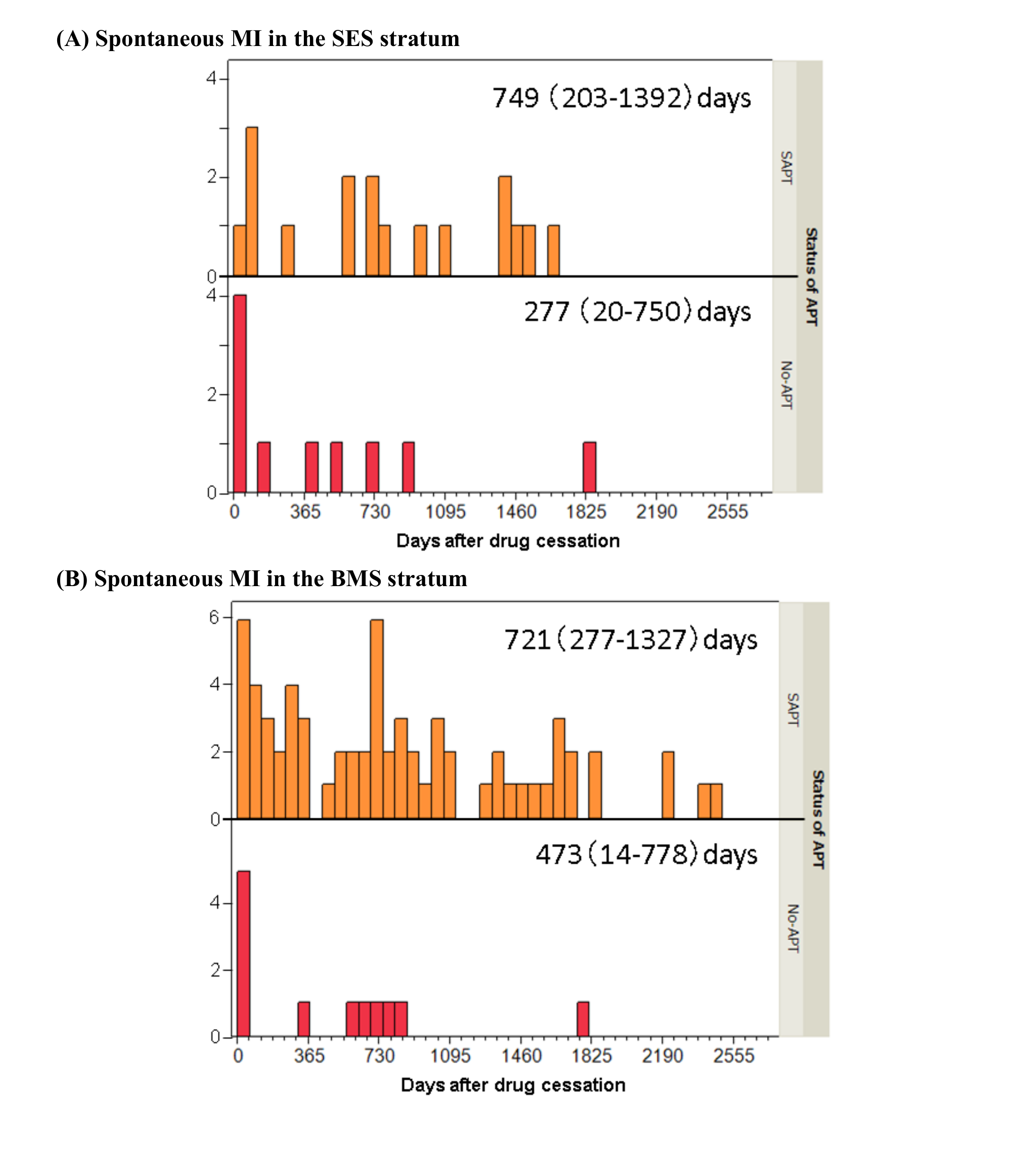

Supplement: S3 Fig — Values are expressed as median (interquartile range) days. APT = antiplatelet therapy, BMS = bare-metal stents, MI = myocardial infarction, SAPT = single-APT, and SES = sirolimus-eluting stents. As for the definitions of the duration, see Table 3 in the manuscript. (TIF) [file pone.0124314.s006.tif]

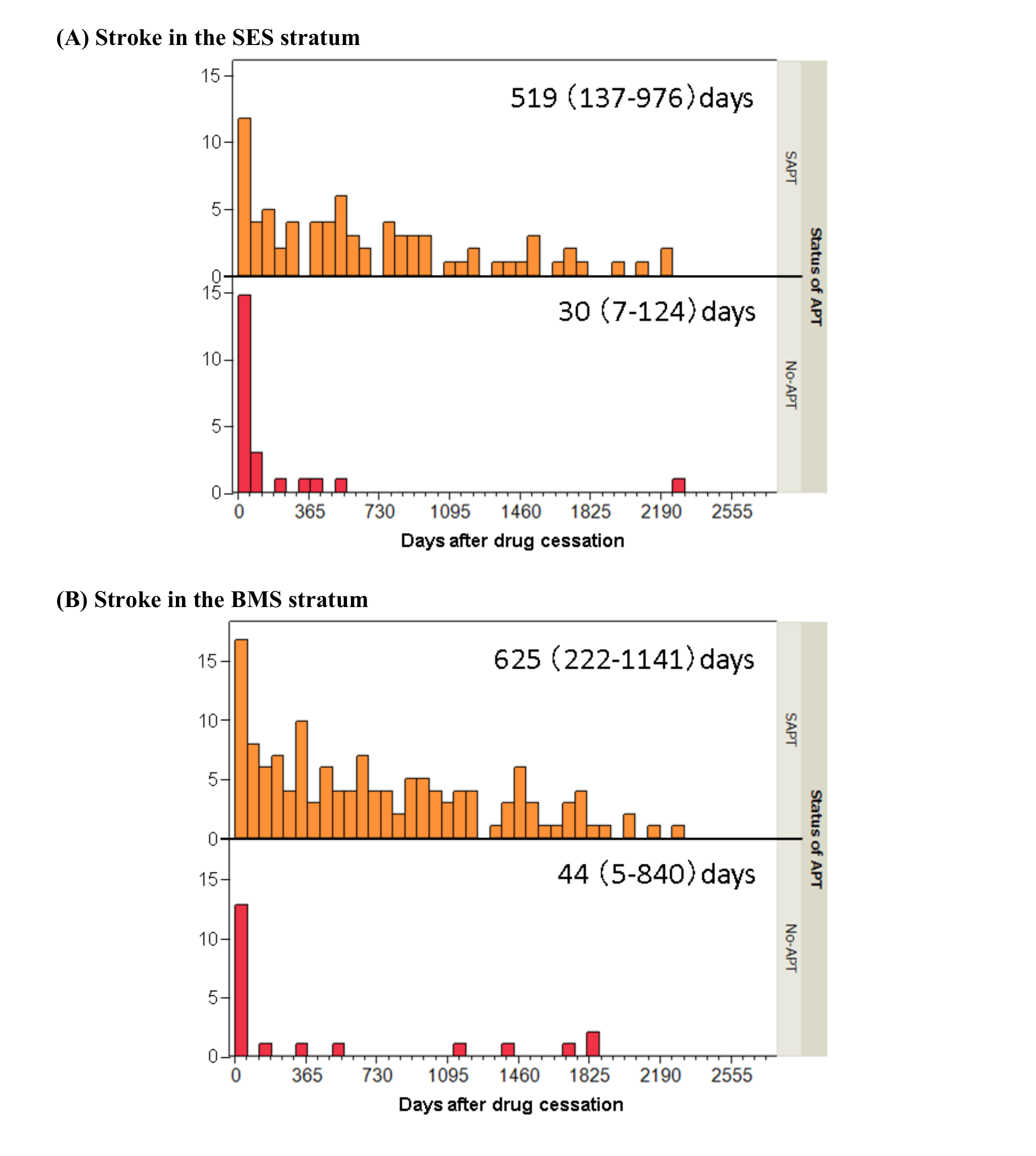

Supplement: S4 Fig — Values are expressed as median (interquartile range) days. APT = antiplatelet therapy, BMS = bare-metal stents, SAPT = single-APT, and SES = sirolimus-eluting stents. As for the definitions of the duration, see Table 3 in the manuscript. (TIF) [file pone.0124314.s007.tif]
